# The Expressive Triad: Structure, Color, and Texture Similarity of Emotion Expressions Predict Impressions of Neutral Faces

**DOI:** 10.3389/fpsyg.2021.612923

**Published:** 2021-02-25

**Authors:** Daniel N. Albohn, Reginald B. Adams

**Affiliations:** Department of Psychology, The Pennsylvania State University, University Park, PA, United States

**Keywords:** face perception, emotion expression, machine learning, impression formation, facial expresions

## Abstract

Previous research has demonstrated how emotion resembling cues in the face help shape impression formation (i. e., emotion overgeneralization). Perhaps most notable in the literature to date, has been work suggesting that gender-related appearance cues are visually confounded with certain stereotypic expressive cues (see Adams et al., 2015 for review). Only a couple studies to date have used computer vision to directly map out and test facial structural resemblance to emotion expressions using facial landmark coordinates to estimate face shape. In one study using a Bayesian network classifier trained to detect emotional expressions structural resemblance to a specific expression on a non-expressive (i.e., neutral) face was found to influence trait impressions of others (Said et al., 2009). In another study, a connectionist model trained to detect emotional expressions found different emotion-resembling cues in male vs. female faces (Zebrowitz et al., 2010). Despite this seminal work, direct evidence confirming the theoretical assertion that humans likewise utilize these emotion-resembling cues when forming impressions has been lacking. Across four studies, we replicate and extend these prior findings using new advances in computer vision to examine gender-related, emotion-resembling structure, color, and texture (as well as their weighted combination) and their impact on gender-stereotypic impression formation. We show that all three (plus their combination) are meaningfully related to human impressions of emotionally neutral faces. Further when applying the computer vision algorithms to experimentally manipulate faces, we show that humans derive similar impressions from them as did the computer.

## Introduction

Decades of research have revealed facial expressions to be a powerful vehicle for social communication. Humans are so tuned to reading dynamic displays from the face that overt expressions tend to influence stable trait impressions (Knutson, 1996). Indeed, years of research suggests that even emotion resembling cues in otherwise neutral faces have a powerful impact on impressions formed (e.g., emotion overgeneralization effect; Zebrowitz et al., 2010). Recent attention has been aimed at training computers to automatically recognize human emotion from the face. These advancements have equipped researchers with a powerful set of tools for exposing new theoretical insights and creating novel societal applications based on this work. Commercially available face reading programs (e.g., FaceReader, Affectiva, and AFDEX) are now widely used across a variety of diverse settings such as classroom observation, user-end experience, human-robot interaction, virtual reality, and marketing.

We know that even faces devoid of overt expression contain a surprising amount of socially relevant information despite being objectively categorized and subjectively posed as neutral (at least in terms of affect). Static physical features such as gender-related appearance and age-related changes in the face alter perceptions and impressions (Zebrowitz et al., 2010; Adams et al., 2016; Albohn and Adams, 2020b). Further, first impressions based on so-called “neutral” faces tend to be consistent across different observers. This suggests that, on some level, individuals are attuned to similar socially relevant cues from which they draw similar judgments. Such judgments are at least in part attributable to the reading of emotion resembling cues that are confounded with gender and age (Adams et al., 2016). The mere resemblance of a face to an expression powerfully influences a wide array of trait impressions of others (Adams et al., 2012). For instance, simply moving the eyebrows to be lower on a non-expressive face leads to greater dominance and anger attributions, whereas moving the eyebrows higher yields greater submissiveness and fear attributions (Keating et al., 1977; Laser and Mathie, 1982). Also, shortening or lengthening the distance between the eyes and mouth results in perceptions of anger and sadness, respectively (Neth and Martinez, 2009). These findings contribute to a growing body of evidence that shows that incidental emotion-resembling cues, and in some cases subtle expressivity lingering on a subjectively non-expressive face, powerfully influence impressions (Zebrowitz et al., 2010; Adams et al., 2016; Albohn and Adams, 2020a).

Various computer vision techniques have complimented the host of findings that suggests information can be derived from non-expressive faces. For instance, Zebrowitz et al. (2007) trained a neural network to detect actual baby vs. adult faces, and then applied this model to detecting such cues in surprise, anger, happy, and neutral expressions. They found that the model detected babyfacedness in surprise expressions, and maturity in anger expressions due to similarities in brow position. Likewise, these researchers later found that both gender and race (Zebrowitz et al., 2010) as well as age (Palumbo et al., 2017) cues in otherwise affectively neutral faces were recognized by the neural network as containing emotion cues. Along these same lines, Said et al. (2009) trained a Bayesian classifier to detect expressions in faces and then applied it to images of neutral faces that had been rated on a number of personality traits (e.g., trustworthy and dominance). Said et al. (2009) found that the trait ratings of the faces were meaningfully correlated with the perceptual resemblance the faces had with certain expressions. These results speak to a mechanism of perceptual overlap whereby expression and identity cues trigger similar processes due simply to their physical resemblance.

## Structure, Color, and Texture Face Metrics

While the past few decades have seen an increase in the development and use of machine learning methods within person perception, most of this work has focused exclusively on evaluating (separately) each metric/feature's influence on model *performance*, rather than how the metric/feature relates back to human visual perception and impression formation. As such, model evaluation is based on absolute performance (i.e., minimizing error from ground truth) rather than attempting to understand how computer vision relates to human vision. Prior research suggests that structure, color, and texture of the face are all important metrics for face identification (Sinha et al., 2006). It stands to reason that each of these metrics has its own influence on human facial emotion recognition as well as downstream impression formation.

Myriad research has examined how facial structural resemblance to emotion expressions relates to impression formation (i.e., emotion overgeneralization). Seminal work has shown that faces with cues that incidentally resemble emotional expressions are subsequently evaluated in terms of that emotional expression (see, e.g., Zebrowitz and McDonald, 1991; Marsh et al., 2005; Zebrowitz et al., 2007). Structural resemblance to a specific expression on a non-expressive (i.e., neutral) face powerfully influences trait impressions of others. In one study, facial structural resemblance to anger expressions was correlated with threatening personality traits (e.g., dominant), and resemblance to happy expressions was correlated with positive traits (e.g., caring; Said et al., 2009). In another study, manipulating neutral faces to structurally resemble anger and fear influenced a whole host of physical, emotional, and person perception impressions, including ones with non-obvious links to emotion such as anger-resembling cues yielding relatively greater impressions of shrewdness and fear-resembling cues yielding relatively greater impressions of intuitiveness (Adams et al., 2012).

Critically, related work has shown that facial structural resemblances to different expressions are related to stereotypes and biases associated with gender. Zebrowitz et al. (2010) first trained connectionist neural network models to discriminate between an expressive (anger, happy, and surprise) face and a neutral face. Next, they applied the trained classifier to neutral faces varying in race and gender. Zebrowitz et al. (2010). In terms of gender, they found that female faces structurally resembled surprise expressions more than male faces. Similarly, male faces structurally resembled anger expressions more than female faces. Finally, male faces structurally resembled happy expressions more than female faces (see, e.g., Becker et al., 2007; Hess et al., 2009; note that this latter finding contrasts with several other prior studies that female faces structurally resemble happy expressions more than male faces). This last point highlights the complex interaction between gender and emotion and suggests that some gendered impressions are influenced from bottom up cues (e.g., metrics of the face), whereas others are overridden by top down stereotypes. In the case of happiness, male faces have been found to structurally resemble happy expressions more than female faces, yet rating studies have shown that more masculine faces are often rated as less trustworthy than feminine faces (see, e.g., Todorov et al., 2008; Adams et al., 2015).

Although less studied than face structure, there is evidence that other features such as color and texture are also important for face identification, impression formation, and emotion judgements (Russell et al., 2006; Sinha et al., 2006). For example, researchers have found that increasing the luminance difference between the eyes and mouth results in more attributions of that face appearing female, while decreasing contrast in the eye and mouth regions result in greater perceptions of the face appearing male (Russell, 2003, 2009). These results are underscored by the observation that women often use cosmetics to increase the contrast of the eyes and lips with the rest of their face and that observers rate women with high facial contrast dimorphism as appearing more attractive (Rhodes, 2006). Face color also appears to have a defining influence on perceptions of religiosity (Rule et al., 2010) as well as judgments of health and attractiveness (Pazda et al., 2016; Thorstenson et al., 2017; Perrett et al., 2020). In both cases, perceptions of “healthier” skin drove impressions and classifications.

Lastly, there has been relatively little work examining the influence of face texture on emotion and impression formation. Most previous research examining how face texture shapes perception has either done so directly by showing that face texture relates to perceived health and attractiveness, or indirectly by showing face cues that are related to skin texture also influence perception (e.g., aging cues). Most research that has examined facial skin texture directly has distinguished it from face skin color. For example, many researchers include in their definition of skin texture components such as skin elasticity (e.g., sagging, wrinkles, smoothness), dermatosis issues (e.g., acne, sun damage, freckles, pores), and facial hair features (e.g., eyebrow thickness). Given that humans are particularly adept at surface property perception (see, e.g., Klatzky et al., 1987), it makes sense that texture would also influence face judgements.

Direct investigations into the influence of surface properties of the face on perception have shown that it is related to perceived health, trustworthiness, and other related traits. Tan et al. (2018) used Gabor wavelet analysis to decompose the texture of a patch of skin on the cheek into three components. They then had participants rate full face pictures on its perceived health. Results showed a significant relationship between perceived health and each Gabor features. Examination of each Gabor feature showed that the three features related to perceived health appeared to be the number of red spots on the skin (less was viewed as healthier), scarring and holes (less perceived as healthier), and roughness (smoother viewed as healthier).

Taken together, facial structure, color, and texture have been shown to be important for both face identification and classification. Individuals appear to use these face cues not only to differentiate faces from non-faces, but also when making judgements, such as the gender or health of a face. All three cues have been shown to both independently and in combination influence emotion expression perception or related personality traits. Critical to the current research, however, is that relatively little research has examined each face metrics' independent and combined contribution to impression formation. Of the work that has examined each metric's contribution, it has done so using either only a subset of features or without the aid of machine learning technology. Thus, there are critical gaps in the literature that the current research attempts to address: What is the relative importance of each face feature for expression classification? How are these features (independently and in combination) related to human-derived impressions? And can they be used to capture subtle or incidental emotion-resemblance on non-expressive faces?

## Overview of Current Work

Previous work has shown that emotion expressions can be predicted via the structure of the face alone, and that a neutral face's structural resemblance to emotion is related to human impressions. However, to the best of our knowledge, no research has extended this work beyond structure, shown meaningful relationships with human impressions beyond just model output, or used the model output to manipulate faces to confirm whether what machine learning algorithms are using to make accurate predictions also drive human impressions. The current work proposes an end-to-end experimental pipeline in which we replicate and extend previous work, while also providing novel experiments to address the current gaps in the literature.

We chose to focus initially on gender-related emotion stereotypes and related trait impressions because these are highly validated and robust effects reported in the literature over the last couple decades (see Adams et al., 2015 for review). Further, recent findings suggest there are visual confounds in emotionally expressive and gender-related appearances cues (Becker et al., 2007; Hess et al., 2009). Thus, the closest to a ground truth to use as an initial test of our algorithm are gender-related emotion and trait impressions. These include the predictions that male neutral faces would be more resembling of anger expressions, which would give rise to more dominant trait attributions, whereas female faces would be more resembling of fear expressions, which would give rise to more affiliative trait impressions. We also predict that, like in previous research, male faces will counter-stereotypically resemble happy expressions more than female faces (Zebrowitz et al., 2010; Adams et al., 2015). Once establishing that, we sought to directly examine the broader theoretical assertion that humans meaningfully utilize emotion-resembling cues in the face when forming impressions by not only showing that computer derived emotion cues mediate human impressions, but by experimentally manipulating faces using algorithmically-derived facial cues and showing that humans utilize those cues to arrive at same impressions predicted by the computer.

**Figure 1:**
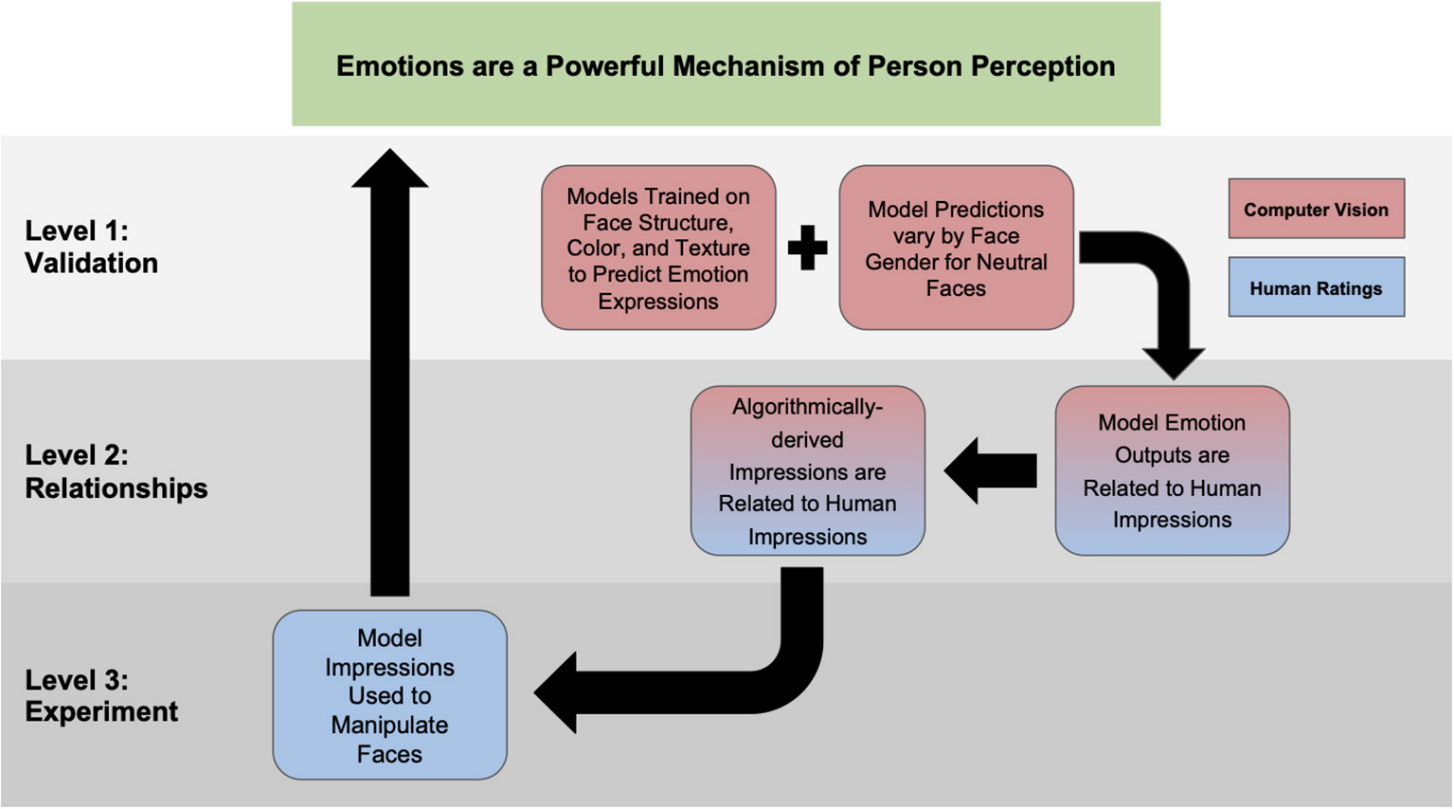
Theoretical roadmap for the current set of studies. Our overall central thesis is presented at the top of the figure. Study 1 corresponds to Level 1; Studies 2 & 3 correspond to Level 2; Study 4 corresponds to Level 3.

We have broken our central thesis into three levels (see Figure 1). The first level (Study 1) attempts to replicate and extend previous work by training models on the structure, color, and texture of the face to see if each was a meaningful predictor of emotion expression. Level 1 also validates our trained models by showing that they predict that male and female neutral faces vary in emotion expressivity in an expected pattern (see Adams et al., 2015). Next, in Level 2, we first replicate previous work and show that our model outputs are meaningfully related to human impressions. More importantly in this study, we also provide novel experimental evidence that the emotion output from our models can be used to algorithmically compute higher-order impressions and that these predict similar human impressions. This finding suggests that humans are, at least in part, using emotions to form their non-emotion, higher-order impressions. Lastly, Level 3 aims to directly test this assertion by taking the trained model outputs and using them to reverse-engineer the actual structure, color, and texture cues that the machine used to derive higher-order impressions (based on its previously trained emotion associations) and show that when faces are altered to resemble these features, humans make similar judgements. By manipulating the faces, we isolate the features available to human participants. Thus, in doing so, we can conclude that any systematic influences on impressions must be driven by the same computer derived emotion-resembling that humans are also using to make their judgements.

In summary, prior research has supported theoretical assertions that humans utilize emotion-resembling cues in neutral faces when deriving higher order impressions. The current work attempts to directly test this supposition, by first showing that computers can use emotion-resembling cues in the face to predict not just human impressions of emotionality, but of higher order person perception. Finally, by systematically manipulating the emotion cues that computers use to arrive at these impressions back into human faces, we aim to isolate the cues available to human impression formation, in order to confirm that the cues computers are using to predict human perception corresponds, at least in part, with the cues humans are using. In doing so, we are able to directly test theoretical assumptions of emotion overgeneralization driving human impressions by using new computer-driven technological advances.

## Study 1

Study 1 applied a machine learning model trained on emotion expressions (see Supplementary Material 1) to neutral faces varying in gender. An additional purpose of Study 1 was to first assess the relative utility of using face metrics beyond structure to predict facial emotion, and then to apply the trained model to neutral faces to assess relative differences in the structure, color, and texture emotion outputs by face gender. Study 1 attempted to better understand previous gender-emotion overgeneralization results by utilizing a broader set of face metrics (in addition to structure) important for emotion classification, namely color, texture, and a combination model. Based on the prior literature, we expected that female faces would be found to resemble fear more than male faces, and that male faces would be found to resemble anger more. Behavioral findings have supported there also being a confound for female faces to resemble happiness more than males (Becker et al., 2007; Hess et al., 2009), but one prior computer vision study found the opposite, so we aimed to further examine this effect here.

## Method

### Model Training

**Figure 2:**
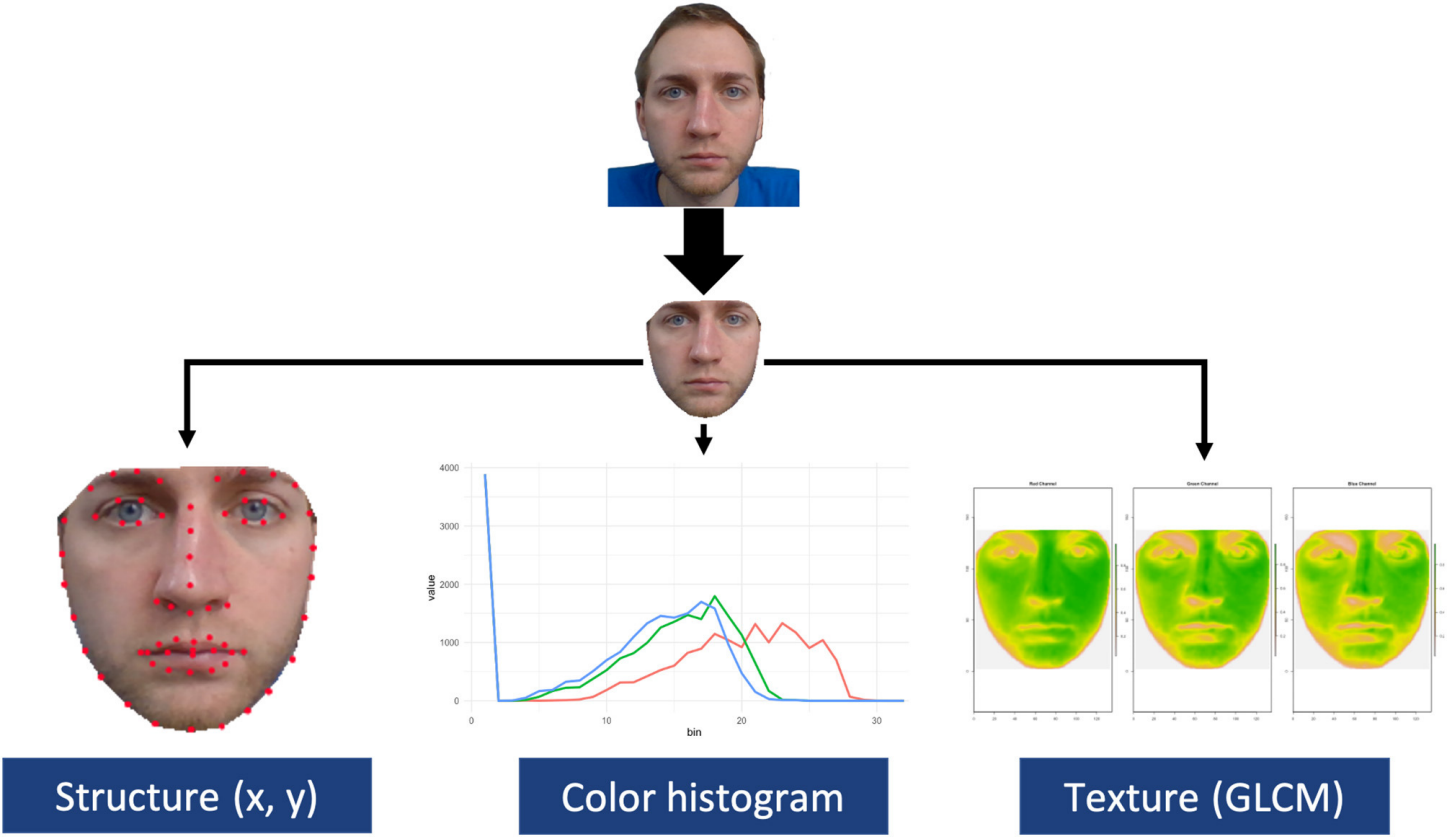
Visual representation of feature extraction of structure, color, and texture from the internal portion of the face.

The full procedure for training the structure, color, texture, and their combination models are reported in Supplementary Material 1. Briefly, structure, color, and texture metrics were extracted from the interior portion of the face for several thousand faces varying in emotion expressivity (see Figure 2). Each model was trained on numerous machine learning algorithms, including a stacked ensemble of multiple models. Each model was assessed via its test accuracy, which was computed on a separate set of faces that each model had not been trained on. The best performing model was retained for each face metric. We also computed a weighted, combined model by summing each model's emotion output weighted by their test accuracy. Each metric model reached a test accuracy that was statistically above chance. However, the weighted, combined model reached an accuracy of nearly 90%, which was statistically higher than all of the other models, χ^2^(0) = 101.49, *p* < 0.001 (see Table 1). This suggests that each face metric uniquely contributed to overall performance. Each model, plus an interactive GUI (see **Figure 6** in Discussion), are available on the first author's website (https://www.daniel-albohn.com/) for research purposes.

**Table 1:** Standardized regression estimates for mediation models presented in Studies 2 & 3.

**Mediation**	**Path estimates (standardized)**				**Indirect effect**	
	**a (se)**	**b (se)**	**c (se)**	**c‘ (se)**	**ab**	**95% CIs**
**STUDY 2**						
Face gender → Anger output → Dominance ratings	0.19^**^ (0.07)	0.32^***^ (0.07)	0.36^***^ (0.07)	0.30^**^ (0.07)	0.06*	[0.01, 0.12]
Anger output → Masculine-Feminine → Dominance Ratings	0.26^***^ (0.06)	0.42^***^ (0.06)	0.38^***^ (0.07)	0.27^***^ (0.06)	0.11*	[0.05, 0.17]
Face gender → Happy output → Trustworthy ratings	0.19^*^ (0.07)	−0.01 (0.07)	−0.36^***^ (0.07)	−0.36^***^ (0.07)	0	[−0.03, 0.03]
Happy output → Masculine-Feminine → Trustworthy ratings	0.19^***^ (0.07)	−0.45^***^ (0.07)	−0.08 (0.07)	0.01^***^ (0.07)	−0.09*	[−0.16, −0.02]
**STUDY 3**						
Face gender → Dominance output → Dominance Ratings	0.24^**^ (0.07)	0.32^***^ (0.07)	0.36^***^ (0.07)	0.29^***^ (0.07)	0.08*	[0.03, 0.14]
Dominance output → Masculine-Feminine → Dominance ratings	0.30^***^ (0.07)	0.41^***^ (0.06)	0.39^***^ (0.07)	0.26^***^ (0.07)	0.12*	[0.06, 0.19]
Face gender → Affiliation output → Trustworthy ratings	−0.12 (0.07)	0.09 (0.07)	−0.36^***^ (0.07)	−0.35^***^ (0.07)	−0.01	[−0.04, 0.01]
Affiliation output → Masculine-Feminine → Trustworthy ratings	−0.16^*^ (0.07)	−0.44^***^ (0.07)	0.13^t^ (0.07)	0.06^***^ (0.07)	0.07*	[0, 0.14]^b^

t *p < 0.1*,

* *p < 0.05*,

** *p < 0.01*,

*** p < 0.001;

b *This CI includes 0 due to rounding*.

### Face Stimuli

White neutral faces varying in gender were taken from the Chicago Face Database (Ma et al., 2015). Neutral faces selected for this study were not used during the training or testing phase of the machine learning models (see Supplementary Material 1). Each individual face was subjected to the feature extraction procedure detailed above, resulting in structure, color, texture, and combined face metrics for each face. The automated feature extraction procedure led to a total sample of 93 male and 90 female neutral faces.

### Computer Vision

For the sake of brevity, we only consider the weighted, combined model results in the main text but consider important results across all three metrics in the Discussion. However, the linear mixed effects regressions and significant pairwise comparisons for the structure, color, and texture individual models, as well as a graphical summary, are presented in Supplementary Material 2.

The weighted, combined model revealed stereotypical gender-emotion associations: Female faces resembled fear [*t*_(1,086)_ = 8.5, *p* < 0.001, CIs [0.09, 0.15]] more than male faces, and male faces resembled anger [*t*_(1,086)_ = −3.8, *p* < 0.001, CIs [−0.08, −0.03]] and happy [*t*_(1,086)_ = −2.63, *p* = 0.009, CIs [−0.06, −0.01]] expressions more than female faces. The full linear mixed effects model results for each metric are presented in Supplementary Material 3.

## Discussion

Study 1 used the trained machine learning models (see Supplementary Material 1) to examine how each face metric was related to a set of neutral faces. The results from Study 1 replicated previous computational and behavioral work examining emotion stereotypes related to gender.

Across structure and texture female faces resembled fear expressions to a greater degree than male faces. Similarly, the structure and texture of male faces resembled anger expressions to a greater degree compared with female faces.

Interestingly, the structure of female faces had slightly greater resemblance to happy expressions, but the color and texture and combination of all three metrics for male faces were more similar to happy expressions compared to female faces. This finding is particularly interesting given stereotypes related to women and smiling. Indeed, a slight smile on a woman is seen as neutral, whereas a similarly intense smile on a man is rated as happy (see, e.g., Bugental et al., 1971). It makes sense that of the three metrics, structure would show the expected gender stereotype of female neutral faces appearing more happy-like since facial landmarks pick up on gross shape information, whereas color and texture capture more nuanced differences (skin tone, aging cues, etc.). For example, if a female's “neutral” face is slightly smiling (i.e., minor upturned corners of the lips), the structure face metric is the most likely candidate to capture this change and use it in the model to make predictions. On the other hand, if someone has stereotypic happy-appearing cues such as “rosy cheeks” or natural “crows' feet” at the corners of their eyes, these are features that color and texture are likely to capture and utilize to make predictions. It is entirely possible that in our test set males had more of these subtle “smile-resembling cues” compared to females.

Overall, the models created replicated and extended previous research when applied to neutral faces varying in gender. Specifically, previous research has shown gender differences with regard to emotion expression resemblance, with male neutral faces more similar to anger expressions and female neutral faces more similar to fear expressions. The current work largely confirms this observation, but across more varied and specific face metrics.

## Study 2

Study 1 examined each model's ability to predict human impressions of neutral faces varying in gender. The goal of Study 2 was to determine the utility of using individual and combined face metrics for predicting subtle emotional content in neutral faces. It is important to examine each model's predictive power on human ratings to assess each model's ecological validity. That is, an accurate machine learning model might classify expressions with a high precision, but it may not be able to classify/predict human responses to the same degree. This is particularly important for the current work as each model was specifically trained on low-level face metrics so that they might be able to detect subtle emotion cues in neutral faces. If each model is correlated with a corresponding and related human impression rating, it suggests that humans are–at least in part–using similar face features to make their judgements about the individual. In addition to examining correlational effects, Study 2 also examined whether structure, color, and texture resemblance to emotions are casual variables through mediation.

We established in the previous study that the trained models predicted the expected outcome of results for faces varying in gender, and thus only present the results from the weighted, combined model in the main text. Further, the weighted, combined model had the highest accuracy out of the three metrics examined, suggesting that it has the most utility in terms of predictive power. However, the results for each individual model (structure, color, and texture) are presented in Supplementary Material 4 and considered in Study 2's Discussion.

## Method

Face stimuli were the same as Study 1.

The Chicago Face Database supplies normed rating data from human participants on a number of different impressions and features. For example, each (neutral) face in the database was rated on how “feminine/masculine” the face appeared. Each neutral face was rated by a minimum of 20 raters (*M* = 43.74). This normed data has been successfully used in recent publications (see, e.g., Hester, 2018) with a high degree of success. The norming data supplied by Ma et al. (2015) in the Chicago Face Database serves as the human impression ratings for Study 2. Of all the suppled norming data, only a subset of emotions and impressions theoretically related to the present work and informed by gender-emotion stereotypes were examined (Johnson et al., 2012; e.g., Adams et al., 2015). Specifically, anger-dominant and happy-trustworthy emotion-impression pairs were examined and interpreted in detail. Further, we examine the relationship between all algorithm emotion outputs and human impressions of dominance, trustworthiness, anger, and happy collapsed across gender via correlations.

Following procedures suggested by previous relevant work (e.g., Zebrowitz et al., 2010), emotion-trait pairs were examined via mediation to see if the machine learning model outputs for the weighted, combined model (and structure/color/texture in the Supplementary Material) mediated the relationship between actual face gender and human impressions. Additionally, we also examined whether perceived masculinity-femininity mediated the relationship between the weighted, combined model emotion output and human ratings. We focused on examining these two mediation models since each explains a different, theoretically important point related to the confounded nature of gender and emotion. Significant findings for the first mediation model (algorithm emotion output as the mediator) would suggest that faces varying in gender have different structure, color, and texture similarity to emotions, and that individuals use these cues that vary by gender to inform their impressions. On the other hand, significant findings for the second mediation model (perceived masculinity-femininity) would suggest that individuals are using structure, color, and texture resemblance to emotion expressions to guide their perceptions of gender, which in turn influence the perceivers' impressions of the face on related impressions.

For each mediation analysis gender was coded as 0 = “Female” and 1 = “Male.” Perceived face gender was computed by taking supplied CFD masculinity and femininity ratings [*r*_(181)_ = −0.96], multiplying the femininity ratings by −1 and adding it to the masculinity ratings such that higher scores on the computed masculine-feminine were indicative of higher masculinity ratings, and lower scores were indicative of higher femininity ratings. All reported mediation indirect effects are estimated with 10,000 bootstrapped samples, and beta coefficients are standardized. Regression coefficients and standard errors for each mediation are reported in Table 1.

## Results

### Dominance

The algorithm output for similarity to anger expressivity partially mediated the relationship between actual face gender and human ratings of dominance, beta = 0.06, CIs [0.01, 0.12]. Neutral male faces appeared more angry-like and faces that appeared more like angry expressions were subsequently rated as higher in dominance.

Similarly, masculine-feminine ratings partially mediated the relationship between algorithm anger output and dominance ratings, beta = 0.11, CIs [0.05, 0.17]. Neutral faces that the algorithm predicted to be more angry-like were rated higher in masculinity, and more masculine appearing faces were rated higher in dominance.

### Trustworthy

The algorithm output for similarity to happy expressivity did not mediate the relationship between actual face gender and human ratings of trustworthiness, beta = 0, CIs [−0.03, 0.03].

However, masculine-feminine ratings mediated the relationship between algorithm happy output and trustworthiness ratings, beta = −0.09, CIs [−0.16, −0.02]. Neutral faces that the algorithm predicted to be more happy-like were rated higher in masculinity, but more masculine appearing faces were rated lower in trustworthiness ratings.

That the machine-derived happy output did not mediate the relationship between face gender and trustworthiness ratings, coupled with the fact that more happy-appearing faces were more masculine is a slightly unexpected finding. These results are discussed more thoroughly in the Discussion.

### Correlations

Correlations between the weighted, combined model and human ratings largely revealed the predicted pattern of results. Machine-derived anger output positively correlated with dominance, masculine-feminine, and anger, and negatively correlated with trustworthy ratings. Interestingly, happy output only correlated with masculine-feminine ratings, with more masculine faces appearing happier. Further, happy output did not correlate with human ratings of happiness. While this may seem odd at first pass, it should be noted that these are correlations with the weighted, combined model.

**Figure 3:**
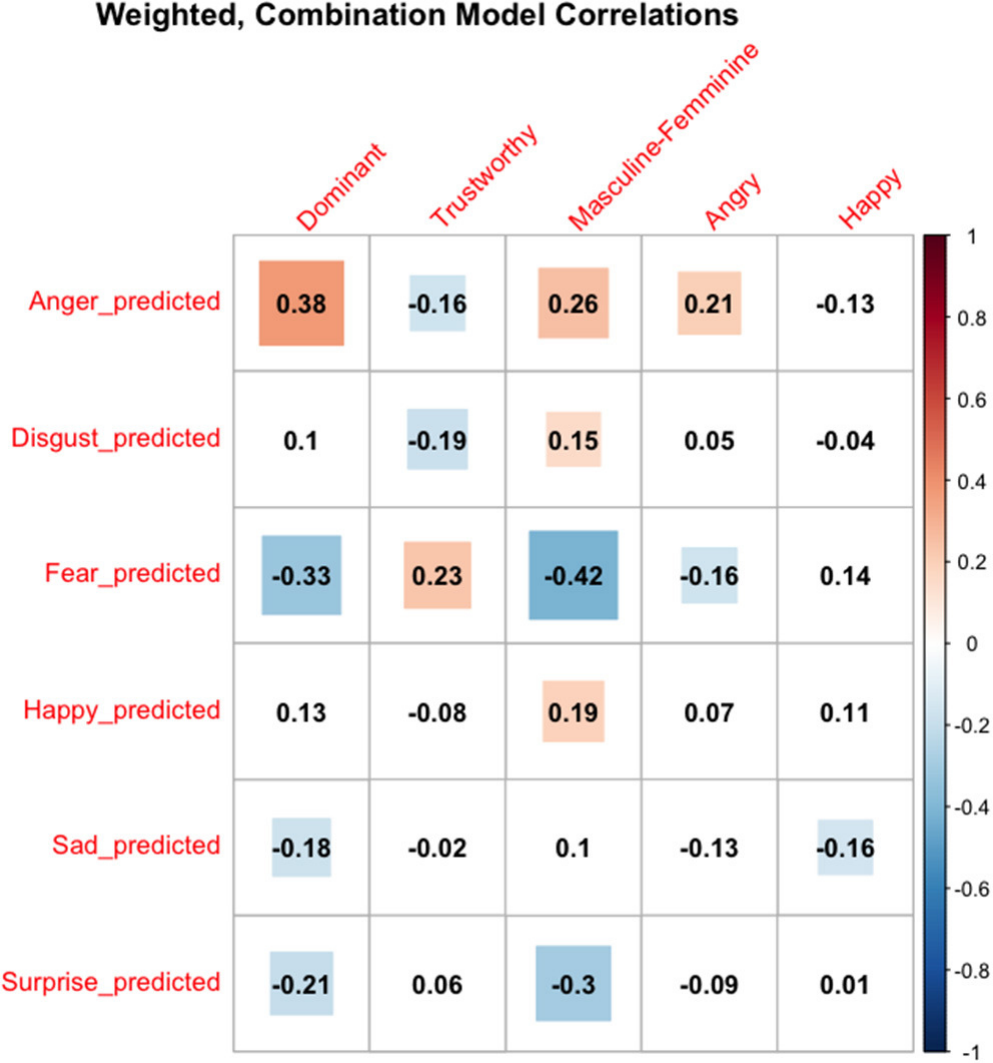
Correlations between weighted combination model predictions and human impressions. Machine-derived output is on the Y-axis, whereas human ratings are on the X-axis. Colored cells are significant. Hot colors are significant positive correlations and cool colors are significant negative correlations.

While not directly related to the present set of studies, significant correlations for the other emotion outputs deserve mention. The more a face was predicted to be expressing disgust, the more masculine and less trustworthy it appeared, mirroring the correlations found for anger output. Similarly, predicted fear output negatively correlated with dominance, masculine-feminine, and anger, while positively correlating with trustworthiness ratings. Finally, sad and surprise output negatively correlated with dominance, while surprise output negatively correlated with masculine-feminine. Taken together, these additional emotion output correlations largely follow the pattern of results expected for gender-emotion stereotypes. Dominance emotions (anger, disgust) positively predict masculinity, dominance, and anger, whereas submissive emotions (fear, surprise) positively predict femininity and trustworthiness. Figure 3 reports all of the correlations.

## Discussion

Study 2 used the trained models from Study 1 to examine the unique and combined ability to predict human-provided trait impressions of neutral faces varying in gender. Only the model output for resemblance to anger expressivity mediated the relationship between actual face gender and human ratings of dominance. Specifically, male neutral faces appeared more anger-like, and more anger-appearing neutral faces were rated higher in dominance. Conversely, happy expression resemblance did not mediate the relationship between face gender and ratings of trustworthiness.

On the other hand, the masculine-feminine ratings mediated the relationship between the model emotion output and human impressions. Specifically, regardless of gender, faces that appeared more angry-like across structure, color, and texture were rated higher in masculinity, and neutral faces rated higher in masculinity were perceived as more dominant. Further, across color, texture, and the weighted model faces that appeared happier were also rated as more masculine, yet the more masculine a face appeared the lower it was rated in trustworthiness. This is in line with previous work, and would suggest that the relationship between happiness and trustworthiness is moderated by actual gender or perceived masculinity-femininity (Adams et al., 2015).

Correlations between the model's anger output and human impressions revealed an expected pattern of results. Anger output positively correlated with dominance, masculine-feminine, anger, and happy ratings, and negatively correlated with trustworthy ratings. On the other hand, resemblance to happy expressions only predicted masculinity ratings.

An examination of the individual correlations for the structure, color, and texture model outputs revealed a similar pattern of results for anger and disgust; they were all correlated with dominant emotions and impressions. However, it was only happy structure that was significantly positively correlated with trustworthiness ratings. Happy color similarity was marginally correlated with happy human ratings, but not with trustworthiness ratings. Finally, happy texture appeared to be correlated with dominant emotions and impressions, much like anger and disgust (see Supplementary Material 4 for the correlation charts for all three metrics).

The observation that faces that resembled happy expressions were rated as more masculine (and in the case of texture dominant-oriented impressions/emotions), yet these same faces were seen as less trustworthy is intriguing and deserves further speculation. These results may be due to an expectancy bias. That is, females are expected to display more happiness than males, and their neutral faces appear more similar to happy expressions. Thus, a female neutral face that appears happy-like across all the metrics examined would be seen as more “neutral” than a similar appearing male face (Fabes and Martin, 1991; Zebrowitz et al., 2010). Indeed, males that have neutral faces that resemble happy expressions might be granted particular attributes *because* they violate expectations. This observation makes logical sense: males who smile (or appear “smiley”) will be rated as appearing more trustworthy than females or males who do not smile. This is consistent with a classic study conducted by Bugental et al. (1971; “Perfidious feminine faces”) in which they found that children perceive their fathers' verbal messages to be friendlier and more approving when delivered with a smile, but no such effect was found for perceptions of their mothers. This finding shows the importance of considering both the phenotypic and stereotypic contributions to face derived trait impressions.

Despite the inconsistent results for happy/trustworthy with the combined model, it should be noted that the expected pattern emerged for the structure mediation model (see Supplementary Material 4). The color and texture output models showed a similar pattern of results as the weighted, combined model, again suggesting a similar confounded gender-emotion effect. Indeed, examining the correlations between happy expression similarity across the three face metrics and each gender revealed that there were few, and negative correlations for females (*r*'s < 0.1, *p*'s > 0.392), but there were meaningful relationships for males. Specifically, there were marginal-to-significant correlations between structure similarity (*r* = 0.14, *p* = 0.182) and color similarity (*r* = 0.27, *p* = 0.009) to happy expressions and human ratings of trustworthiness. These relationships only being present for males strengthens our argument that males may indeed by granted counter-stereotypic traits at a higher rate than females simply due to their gender.

Taken together, these results largely conform with previous work that has shown similar emotion-overgeneralization results between face gender and structural similarity to emotion expressions (e.g., Zebrowitz et al., 2010). However, these results extend previous work by showing that there is a causal relationship between the face metric (structure, color, and texture) similarity to expressions and gendered impressions.

Overall, the models created in Study 1 replicated and extended previous research that has shown actual and perceived gender differences with regard to emotion expression expectations and expression resemblance, with the most robust effect in our data being that male neutral faces were more similar to anger expressions, and thus rated as more dominant. The predicted relationships between emotion expression resemblance and impressions also occurred, with the largest effects seen for anger and disgust similarity increasing power-oriented impressions, and fear and surprise resemblance increasing attributions of submission impressions. In sum, results across neutral face similarity to specific emotions and its relationship to human impressions replicates and extends past work.

## Study 3

Study 2 showed that the emotion output from the trained models were related to human impressions, and in the case of dominance was even causal in explaining the relationship between gender and stereotypic human impressions. Given the significant findings of Study 2, Study 3 aimed to show that higher-order impressions can be algorithmically computed from the emotion output of the machine learning models and that these algorithmically-computed impressions are related to human impressions.

Research across multiple decades suggests that two powerful dimensions of human impression formation are dominance/power and affiliation/trustworthiness. For example, Knutson (1996) extended Wiggins' interpersonal circumplex (Wiggins et al., 1988) to show that emotion expressions fall within a two-dimensional dominance/affiliation face space. Later, Todorov et al. (2008) showed that specific traits could be represented within a two-dimensional dominance and trustworthy face space. Further, Todorov et al. (2008) showed that computer-generated neutral faces at the extremes of the trustworthy dimension mimicked expressive features: +3 SD trustworthy neutral faces appeared happy, and −3 SD trustworthy neutral faces appeared angry. Given the importance of these dimensions to person perception research, Study 3 focuses on the impressions of dominance and trustworthiness/affiliation.

## Method

Stimuli were the same male and female neutral faces used in Study 2 from the CFD face database (Ma et al., 2015).

Human impressions used in Study 3 were the same as those used in Study 2 (i.e., provided by the CFD face database). The machine-derived impressions were computed from the emotion output of the weighted, combined model detailed in Study 1 (see below).

Dominance and affiliation were algorithmically computed for each neutral face and derived from the emotion resemblance metrics. Estimation of dominance and affiliation was accomplished by following a similar procedure reported by Knutson (1996), but in reverse. Whereas, Knutson (1996) computed the spatial location of each emotion expression in dominance/affiliation face space, here the opposite approach was taken. Specifically, the face space emotion expressions scores found by Knutson (1996) (see Table 2) were multiplied by the emotion expression output from the weighted, combined machine learning model built in Study 1 to algorithmically project each neutral face onto a two-dimensional dominance by affiliation social face space.

**Table 2:** The relative dominance and affiliation values for each emotion expression found by Knutson (1996).

**Face space**	**Anger**	**Disgust**	**Happy**	**Fear**	**Sad**	**Surprise**
Dominance	1	0.6	1	−0.5	−1	−0.5
Affiliation	−1.5	−1	2	0.5	0.1	0.1

Specifically, dominance, $\hat{D}$, was computed by multiplying the emotion expression value by the dominance value (y axis) found by Knutson (1996), *Y*_*d*_, with the weighted, combined model emotion output, *I*_*j*_, and summing across all emotions, *i*, such that

$$\begin{array}{l} \hat{D}=\underset{i=1}{\sum }Y_{id}*I_{ij} \end{array}$$

Similarly, affiliation, $\hat{A}$, was computed by multiplying the emotion expression value by the affiliation value (x axis) found by Knutson (1996), *X*_*a*_, with the weighted, combined model emotion output, *I*_*j*_, and summing across all emotions, *i*, such that

$$\begin{array}{l} \hat{A}=\underset{i=1}{\sum }X_{ia}*I_{ij} \end{array}$$

## Results

Like Study 2, algorithmically computed dominance and affiliation were assessed to see if they mediated the association between human ratings of dominance/trustworthiness and gender, and if perceived masculinity-femininity mediated the relationship between the algorithmically-derived impressions and human ratings. Table 1 presents the standardized regression coefficients for each mediation.

### Dominance

Algorithmically computed dominance scores partially mediated the relationship between face gender and dominance ratings, beta = 0.08, CIs [0.03, 0.14]. Neutral male faces appeared more dominant and as faces appeared more dominant-like within face space they were subsequently rated as higher in dominance.

Similarly, human masculine-feminine ratings partially mediated the relationship between algorithmically computed dominance scores and human ratings of dominance, beta = 0.12, CIs [0.06, 0.19]. Regardless of actual gender, faces that were higher on algorithmically-derived dominance were perceived by humans to be higher in masculinity, and faces higher in masculinity were perceived by humans to be higher in dominance.

### Affiliation

Algorithmically computed affiliation scores did not mediate the relationship between face gender and trustworthy ratings, beta = −0.12, CIs [−0.04, 0.01]. However, human ratings of masculinity-femininity did mediate the relationship between algorithmically computed affiliation scores and human ratings of trustworthiness, beta = −0.16, CIs [0.003, 0.14]. Specifically, regardless of actual face gender, faces that were higher on computed affiliation were seen as less masculine, and more masculine-appearing faces were rated lower on trustworthiness. In other words, faces that were higher in computed affiliation were seen by humans as more feminine, and more feminine faces were rated as overall more trustworthy.

## Discussion

Study 3 showed that the emotion output from the machine learning models could be used to algorithmically derive higher-order impressions that were meaningfully related to similar human impressions. Indeed, algorithmically computed dominance scores mediated the relationship between face gender and human ratings of dominance. Further, human ratings of masculine-feminine mediated the relationship between the machine outputs of dominance and affiliation, and dominance and trustworthiness, respectively. Together with the results from Study 2, this suggests that humans are partially using facial metric features in the face to derive their impressions of dominance and trustworthiness ratings of neutral faces. This conforms with research that suggests emotion expressions are a powerful mechanism that individuals use to form impressions of others, particularly when other information is absent, as is the case with neutral faces (see, e.g., Zebrowitz et al., 2010; Adams et al., 2012; Albohn et al., 2019; Albohn and Adams, 2020a).

One reason why algorithmically computed affiliation did not mediate the relationship between face gender and trustworthiness may be due to how the scores were algorithmically derived. That is, the dominance model had matching algorithmically computed and human impressions, whereas the affiliation model had algorithmically computed affiliation scores but human trustworthiness scores. While affiliation and trustworthiness are highly correlated (and in some cases used interchangeably), this may have added additional noise to the model. It is also possible, given the findings of Study 1 and 2 whereby masculinity appears to be related to perceptions of masculinity, that the affiliation effects based on emotion are more tenuous. Future work should account for this shortcoming by algorithmically calculating trustworthy scores or having humans rate faces on affiliation. Similarly, stronger models may allow for more nuanced relationships to emerge.

## Study 4

Study 2 showed the relative and combined importance of each face metric on various human-provided impressions, and Study 3 showed that the emotion output from the machine learning models could be used to algorithmically estimate higher order impressions. Study 4 attempted to extend these results by using an experimental design through which neutral faces were psychophysically manipulated to appear more or less like a specific impression in a systematic manner. The goal of the psychophysical manipulation was to physically manipulate the face stimuli with explicit intention of creating psychological changes in the subjective perception of the participants.

Study 4 aimed to show that higher-order impressions can be algorithmically computed from the emotion output of machine learning models and that these algorithmically-computed impressions are related to human impressions. Without direct comparisons across trait dimensions and within face identity, one cannot make any conclusions about whether it was the individual face features (as assessed via machine learning) that drive human impressions formation.

It was predicted that when a neutral face is transformed to appear more like a neutral face that has been classified by a machine learning algorithm into dominance/affiliation face space as based on resemblance to specific expressions (e.g., angry/happy) it will be rated by humans in a similar manner based on the same physical properties (structure, color, texture) of the face that the machine used to make its classification.

## Method

Study 4 manipulated faces to appear more like faces that would appear in Wiggins' circumplex using dominance and affiliation as criterion for this procedure. Again, dominance and affiliation were selected due to the fundamental nature of these attributes on person perception (Knutson, 1996; Todorov et al., 2008).

Using dominance-affiliation face space has the added benefit of each quadrant of the face space roughly corresponding to a specific and dissociable impression that can then be derived from the face. For example, the upper-left quadrant corresponds to a high dominance, low affiliative face and these faces are often rated as appearing angry (for review and examples see, Knutson, 1996).

### Participants

A total of 216 (91 male, 122 female, one gender nonconforming, 2 other; *M* = 20.05, *S*D = 4.19) undergraduate participants participated online in the study in exchange for course credit. Table 3 reports participant race and ethnicity. Participants were allowed to select all that applied.

**Table 3:** Participant demographics for Study 4.

**Race**	**Latinx/Hispanic**	**Not latinx/Hispanic**	**Not reported**
Asian	0	20	0
Black	2	10	0
Black, White, Asian, Native American/American Indian	0	1	0
White	8	158	0
White, Asian	0	6	0
White, Asian, Native Hawaiian/Pacific Islander	0	1	0
White, Native American/American Indian	1	0	0
White, Other	1	0	0
Native American/American Indian	0	1	0
Not Reported	0	0	1
Other	2	4	0

### Stimuli and Transformation Procedure

White neutral faces were extracted from the CFD (Ma et al., 2015), FACES (Ebner et al., 2010), NIMSTIM (Tottenham et al., 2009), RAFD (Langner et al., 2010), MR2 (Strohminger et al., 2016), and FACES (Minear and Park, 2004) databases resulting in a total of 446 neutral faces. Each neutral face was subjected to the same pipeline as reported in Study 1 & 3 to calculate the predicted emotion expression classification values using the weighted, combined model and dominance and affiliation scores.

**Figure 4:**
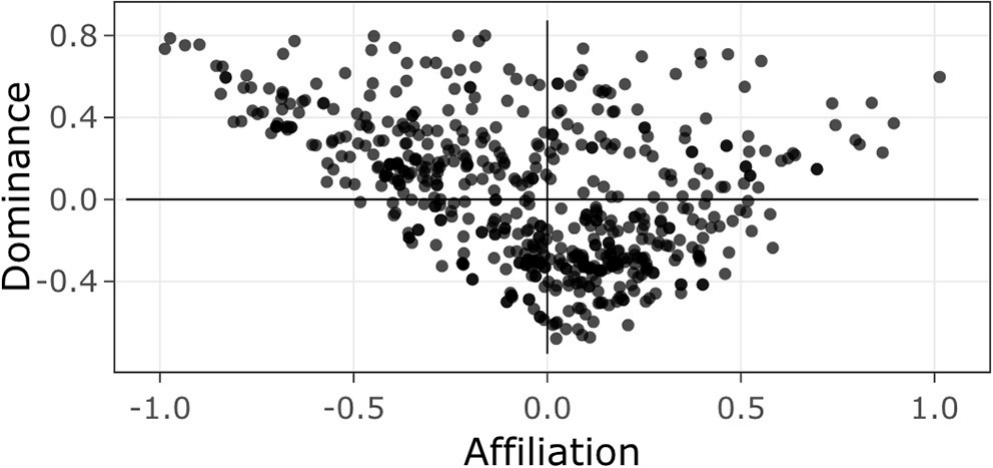
Distribution of neutral faces in computed dominance (y) and affiliation (x) social face space.

After dominance and affiliation scores were computed for each face, this information was used to calculate each faces' distance and angle from the origin in face space. Euclidean distance, $\hat{E}$, was calculated as

$$\begin{array}{l} \hat{E}=\sqrt{{\left(x_{2}-x_{1}\right)}^{2}+{\left(y_{2}-y_{1}\right)}^{2}} \end{array}$$

where *x*_2_ and *y*_2_ were set to the origin, or (0, 0), and *x*_1_ and *y*_1_ were set as affiliation and dominance values, respectively. The angle from the origin, θ, was calculated by first computing the radians from the arctangent function via

$$\begin{array}{l} \theta =atan2\left(y,\;x\right) \end{array}$$

where *x* and *y* were each faces' affiliation and dominance values, respectively, and then converted to degrees. Figure 4 shows the distribution of faces within each quadrant.

Faces were selected for the transformation procedure following a stepwise process. First, four male (*M* = 0.14) and four female (*M* = 0.08) neutral faces from the CFD database were selected by locating faces that had the shortest distance from the origin ([0, 0] in computed face space). Next, these eight faces were morphed with a random subset of neutral faces that fell within the middle range of each quadrant of the calculated face space.

Each of the eight neutral faces was morphed with a randomly selected neutral face from each quadrant. This process was repeated four times for each of the eight faces, resulting in 240 total neutral faces. Faces were selected from the larger set if 1) they had a distance from the origin >0.15, and 2) fell within the middle portion of each quadrant based on their angle from the origin. These parameters were adopted in order to guarantee that each face fell within a reasonable position within the computed face space (i.e., not too close to the origin, or near the edges of the face space quadrant). Specifically, faces in quadrant one (upper right) were between 10 and 80°. Faces in quadrant two (upper left) were between 100 and 170°. Faces in quadrant three (lower left) were between 190 and 260°. Faces in quadrant four (lower right) were between 190 and 350°.

After each neutral face was classified into a quadrant, the top 20 male and 20 female neutral faces from each quadrant were placed into a pool to be randomly selected for transformation with the close-to-origin neutral faces identified in the previous step. Similarly, the top 20 anger and 20 joy neutral faces were acquired from the full set of images by taking the highest rated neutral faces for each expression and gender.

**Figure 5:**
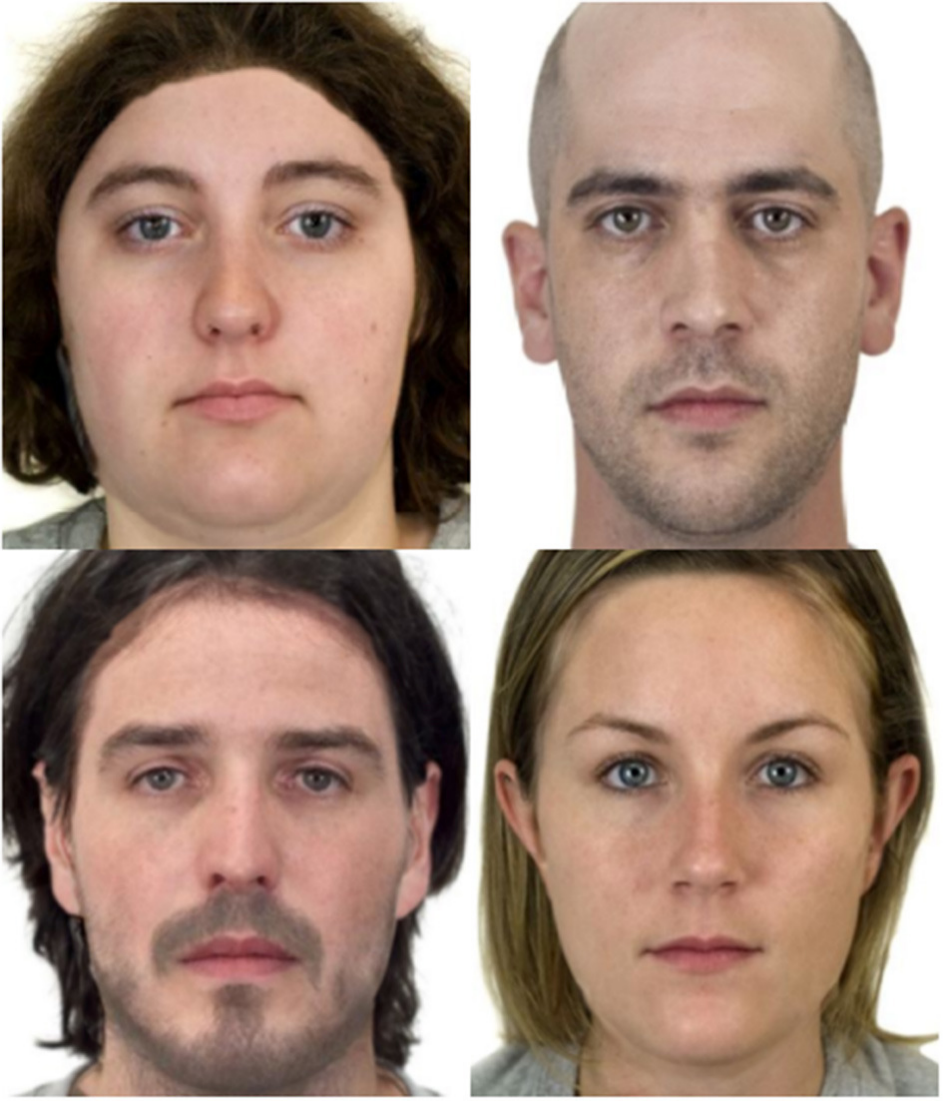
Examples of transformed stim rated for dominance (top) and trustworthiness (bottom). Left column depicts transforms rated low in the trait. Right column depicts transforms depicted high in the trait.

The structure, color, and texture of the randomly selected images from each quadrant, as well as anger and happy, were transferred onto the close-to-origin neutral faces at a 50–50 split using PsychoMorph v.6 (see Tiddeman et al., 2005). Close-to-origin and randomly selected neutral faces were gender matched before each transform. After transformation, the faces were cropped to a standardized size and visually inspected for artifacts. Unrealistic appearing images were manually manipulated to appear more genuine or were discarded. Manual inspection of the images reduced the final set of manipulated and morphed images to 221. Example morphed images are shown in Figure 5.

### Participant Procedure

Participants completed the rating portion of the study using an online participant recruitment platform run by the authors' host University. Participants were randomly presented with 70 faces from the full set of 221 faces. Each face was presented focally with a Likert-type scale underneath the image ranging from “1—Not at all” to “7—Very much.” Participants were asked to rate each face on how much each person appeared “angry,” “happy,” “trustworthy,” “dominant,” “healthy,” “attractive,” “babyish,” and “smart.” At the beginning of the experiment participants read instructions that included a short definition of each trait. Each trial consisted of rating each face on all of the traits before moving onto the next transformed image. Individual ratings were separated by a 100 ms fixation cross. Ratings for each stimulus were randomized between stimuli, and participants were instructed when a new face rating block was about to occur. Participants then filled out basic demographic information before debriefing and returned to the online participation platform.

## Results

Only the results for the four quadrants are presented in the main text. Results for anger and happy psychophysical transforms are presented in Supplementary Material 5 but considered in the Study Discussion.

### Data Preprocessing and Analysis Strategy

Due to the nature of online studies (e.g., lack of accountability, inattention, etc.), some preprocessing of the data was necessary. First, participants were dropped from analyses if their responses had little variability across all of the trials, specifically a standard deviation <0.4 (*n* = 2). Second, individual participant trials were eliminated if the trial reaction time was <50 ms (<6.6%) or >10,000 ms (<1.8%). These two elimination criteria ensured that only reliable responses from participants who were paying attention were analyzed. Additionally, 1 was serially subtracted from each participants' individual trial response so that responses ranged from 0 to 6, allowing for greater interpretability.

Results are reported as separate linear mixed-effects regression models for each quadrant because analyzing a full model that contained every possible comparison (six quadrants by seven trait ratings by two face genders) would result in 84 comparisons and significantly reduce power. Each model compares ratings of the original, close-to-origin neutral face with ratings for a specific quadrant. The models include fixed effects for each block/valence (i.e., impression rating) and stimulus gender and random effects for each participant. Lastly, the models include each image's average attractiveness ratings as covariates (see, Zebrowitz et al., 2010). Supplementary Material 6 reports the full linear mixed effects regressions for each quadrant.

### Quadrant I: High Dominance, High Affiliation

As expected, there was a main effect of rating, *F*_(6,22183.79)_ = 102.11, *p* < 0.001, η^2^= 0.41. No other main effects were significant. However, there were significant interactions. First, there was an interaction between face type and rating [*F*_(6,22183.79)_ = 6.33, *p* < 0.001, η^2^= 0.03]. Quadrant I neutral transforms were rated higher than close-to-origin neutral faces on dominance, estimate = −0.19, *SE* = 0.08, *t*_(162.76)_ = −2.25, *p* = 0.026, CIs [−0.35, −0.02], and happy, estimate = −0.19, *SE* = 0.08, *t*_(162.76)_ = −2.3, *p* = 0.023, CIs [−0.36, −0.03]. Quadrant I faces were also rated lower on babyishness, estimate = 0.28, *SE* = 0.08, *t*_(162.76)_ = 3.37, *p* < 0.001, CIs [0.12, 0.45].

There was also a significant interaction between impression rating and gender [*F*_(6,22183.79)_ = 17.31, *p* < 0.001, η^2^= 0.07]. Female faces were rated higher than male faces on babyishness, estimate = 0.34, *SE* = 0.08, *t*_(163.13)_ = 4.08, *p* < 0.001, CIs [0.18, 0.5], and trustworthiness, estimate = 0.17, *SE* = 0.08, *t*_(163.13)_ = 2.02, *p* = 0.045, CIs [0, 0.33]. Male faces were rated higher in dominance than female faces, estimate = −0.5, *SE* = 0.08, *t*_(163.13)_ = −6.03, *p* < 0.001, CIs [−0.67, −0.34].

Finally, there was a three-way interaction between face type, impression rating, and gender [*F*_(6,22183.79)_ = 4.91, *p* < 0.001, η^2^= 0.02]. Examining this three-way interaction revealed a number of gender-specific interactions. Specifically, quadrant I female faces were rated as less babyish than close-to-origin neutral faces, estimate = 0.35, *SE* = 0.12, *t*_(159.4)_ = 3, *p* = 0.003, CIs [0.12, 0.58]. Quadrant I female faces were also rated as happier [estimate = −0.32, *SE* = 0.12, *t*_(159.4)_ = −2.75, *p* = 0.007, CIs [−0.55, −0.09]], healthier [estimate = −0.23, *SE* = 0.12, *t*_(159.4)_ = −1.93, *p* = 0.056, CIs [−0.46, 0.01]], and (marginally) more trustworthy [estimate = −0.23, *SE* = 0.12, *t*_(159.4)_ = −1.93, *p* = 0.055, CIs [−0.46, 0]] than their close-to-origin counterparts. Male quadrant I faces were only rated as more dominant than close-to-origin neutrals, estimate = −0.28, *SE* = 0.12, *t*_(166.16)_ = −2.34, *p* = 0.021, CIs [−0.51, −0.04].

### Quadrant II: High Dominance, Low Affiliation

There was a main effect of rating, *F*_(6,22652.59)_ = 95.24, *p* < 0.001, η^2^= 0.44. No other main effects were significant. However, there was a significant interaction between rating and gender [*F*_(6,22652.59)_ = 18.73, *p* < 0.001, η^2^= 0.09]. Female faces were rated higher than male faces on babyishness, estimate = 0.36, *SE* = 0.09, *t*_(144)_ = 4.08, *p* < 0.001, CIs [0.18, 0.53], and health, estimate = −0.29, *SE* = 0.09, *t*_(144)_ = −3.32, *p* < 0.001, CIs [−0.46, −0.12]. Male faces were rated higher in dominance, estimate = −0.53, *SE* = 0.09, *t*_(144)_ = −6.11, *p* < 0.001, CIs [−0.71, −0.36].

While there was not a three-way interaction between face type, rating, and gender [*F*_(6,22652.59)_ = 0.52, *p* = 0.796, η^2^= 0], *post hoc* exploratory analyses were still computed and analyzed. As expected, male quadrant II transforms were rated higher on dominance than close-to-origin male faces, estimate = −0.3, *SE* = 0.12, *t*_(146.35)_ = −2.42, *p* = 0.017, CIs [−0.55, −0.06]. No other pairwise comparisons reached significance.

### Quadrant III: Low Dominance, Low Affiliation

There was a main effect of rating, *F*_(6,21041.39)_ = 85.26, *p* < 0.001, η^2^= 0.42. No other main effects were significant. There was a significant interaction between rating and gender [*F*_(6,21041.39)_ = 25.28, *p* < 0.001, η^2^= 0.13]. Female faces were rated higher than male faces on babyishness, estimate = 0.53, *SE* = 0.08, *t*_(201.08)_ = 6.81, *p* < 0.001, CIs [0.38, 0.69], and health, estimate = −0.22, *SE* = 0.08, *t*_(201.08)_ = −2.76, *p* = 0.006, CIs [−0.37, −0.06]. Male faces were rated higher in dominance, estimate = −0.52, *SE* = 0.08, *t*_(201.08)_ = −6.6, *p* < 0.001, CIs [−0.67, −0.36].

Finally, there was a three-way interaction between face type, rating, and gender (*F*_(6,21041.39)_ = 2.53, *p* = 0.019, η^2^= 0.01). Quadrant III female faces were marginally rated as more happy than close-to-origin neutral faces, estimate = −0.19, *SE* = 0.11, *t*_(197.7)_ = −1.73, *p* = 0.085, CIs [−0.41, 0.03]. Quadrant III male faces were only rated as angrier [estimate = −0.22, *SE* = 0.11, *t*_(203.62)_ = −1.97, *p* = 0.05, CIs [−0.44, 0]] and more dominant [estimate = −0.26, *SE* = 0.11, *t*_(203.62)_ = −2.38, *p* = 0.018, CIs [−0.48, −0.05]] than their close-to-origin counterparts.

### Quadrant IV: Low Dominance, High Affiliation

For quadrant IV faces there was a main effect of rating, *F*_(6,21871.16)_ = 91.05, *p* < 0.001, η^2^= 0.4. No other main effects were significant. However, there was a significant interaction between rating and gender [*F*_(6,21871.16)_ = 17.91, *p* < 0.001, η^2^= 0.08]. Female faces were rated higher than male faces on babyishness, estimate = 0.39, *SE* = 0.09, *t*_(152.34)_ = 4.52, *p* < 0.001, CIs [0.22, 0.56], and marginally higher on trustworthiness, estimate = 0.14, *SE* = 0.09, *t*_(152.34)_ = 1.66, *p* = 0.099, CIs [−0.03, 0.31]. Male faces were rated higher in dominance, estimate = −0.49, *SE* = 0.09, *t*_(152.34)_ = −5.71, *p* < 0.001, CIs [−0.66, −0.32], and marginally higher on health, estimate = −0.17, *SE* = 0.09, *t*_(152.34)_ = −1.96, *p* = 0.052, CIs [−0.34, 0].

Finally, there was a three-way interaction between face type, rating, and gender [*F*_(6,21871.16)_ = 3.14, *p* = 0.004, η^2^= 0.01]. Examining this three-way interaction revealed one significant pairwise comparison: Quadrant IV female faces were rated as healthier than close-to-origin neutral faces, estimate = −0.24, *SE* = 0.12, *t*_(149.02)_ = −1.98, *p* = 0.049, CIs [−0.48, 0].

## Discussion

Study 4 experimentally and psychophysically manipulated the structure, color, and texture of neutral faces with other faces that were reliably and highly categorized by the weighted, combined model as resembling a specific expression or a given trait (within an estimated two-dimensional face space model based on dominance and affiliation). It was predicted that when a neutral face was experimentally manipulated to structurally, color-wise, and texturally resemble a different neutral face that highly resembled an expression/trait that it would be perceived in much the same manner as if it had an overt expression or was explicitly rated as high in that trait.

Results across four quadrants within the face-space dimension largely supported the predicted pattern of results. Specifically, quadrant I transform faces (high dominant, high affiliative) were rated as more dominant, happy, and less babyish than unaltered neutral faces. These results were largely driven by male transforms being rated as more dominant, and female faces being rated as happier and less babyish. These results largely follow from the observation that quadrant I faces typically appear more “smiley” and appear to have healthy skin color (carotenoids; see, e.g., Perrett et al., 2020). These effects are further corroborated by the results from the happy transforms (see Supplementary Material 6). Happy transforms–particularly female faces–were rated as healthier, more trustworthy, and more intelligent. These results follow previous research that shows that individuals who express positive emotions are endowed with more positive traits (e.g., the halo effect).

Quadrant II male transforms (high dominance, low affiliative) were rated as more dominant than unaltered faces. Similarly, male anger transforms were rated as more dominant and less babyish than unaltered neutral faces (see Supplementary Material 6). It appears that the structure, color, and texture of neutral faces that appear more anger-like influence ratings of dominance more than directly changing ratings of anger. This may be due to the fact that making an emotion expression judgment about a neutral face is harder than making a higher order impression judgment. That is, rating a neutral appearing face on dominance is easier for participants than rating a neutral face on how “angry it appears” because–by virtue of its definition–a neutral face is low in emotional expressivity.

Quadrant III male transforms (low dominance, low affiliation) were rated as angrier and more dominant than unaltered neutral faces. This pattern of results is not entirely unexpected, as male faces are more dominant to begin with. Thus, observers might only be using (negative) valence information to make judgements about the faces. Lastly, quadrant IV female transforms (low dominance, high affiliation) were rated as less healthy than unaltered neutral faces.

Despite significant results, there are still a number of limitations that deserve discussion. One unexpected finding was that anger transforms were not rated as less trustworthy than unaltered neutral faces. This may be due to the fact the faces chosen were less trustworthy to begin with before manipulation. Indeed, neutral faces are often rated as more negative to begin with (Lee et al., 2008). Again, this is underscored by the observation that only happy transforms were rated as significantly higher in trustworthiness. Further, re-running the current analyses with more stimuli or fewer, more direct comparisons could raise the power of each model. This would help to raise the significance of marginally significant comparisons, or non-significant comparisons that are in the correct and predicted direction. Indeed, male anger transforms were rated as numerically *less* trustworthy than close-to-origin neutral faces, yet this comparison failed to reach significance [estimate = −0.13, *SE* = 0.12, *t*_(138.34)_ = −1.08, *p* = 0.282, CIs [−0.37, 0.11]].

## General Discussion

The present research proposed an extension of previous computer vision work that has examined the structural resemblance of neutral faces to specific expressions and personality traits (Said et al., 2009; Zebrowitz et al., 2010). While previous work has examined the utility (Figure 1, Level 1) and correlational relationships (Figure 1, Level 2) between machine learning output and human impressions, no work to our knowledge has taken machine learning output and constructed higher-order impressions (Figure 1, Level 2) or used that output to manipulate faces to experimentally show that humans are using similar metrics as machines to form impressions (Figure 1, Level 3), let alone an end-to-end experimental pipeline. To this end, our work adds novel insight into a growing body of literature which shows that emotion expressions are a powerful mechanism of person perception (see Table 4 for a summary of findings and significance).

**Table 4:** Summary of findings and significance across the four studies presented in the current research.

**Study**	**Finding**	**Significance**
1	Face **structure**, **color**, and **texture**, and their **weighted combination** are reliable predictors of facial affect; each metric varies by gender in an expected manner	**Validates** model; **extends previous work** showing gender differences in facial structure to texture and color as well
2	All three metrics **correlate** with, and in some cases, **mediate the relationship** between face gender and **human impressions**	Provides correlational evidence that the **metrics used by machine learning** to predict emotions **relates to human impressions** in an expected manner
3	**Algorithmically-derived** impressions of dominance and affiliation are related to **human impressions** of dominance and trustworthiness	Demonstrates that **higher-order impressions can be derived from machine learning** output trained on emotions
4	**Algorithmically-derived** impressions can be used to **reverse-engineer** important **structure, color**, and **texture** features in neutral faces	**Experimentally demonstrates** that metrics machine learning models use to **predict emotions** are also **used by humans** to **form impressions**

Across four studies we replicated and extended prior work by showing that similarities in structure, color, and texture (as well as their weighted combination) to expressions vary across neutral facial appearance associated with actual and perceived face gender in a largely stereotypic manner. Further, this work provides evidence that all three face metrics examined (plus their combination) predict human impressions of emotionally neutral faces similar to what would be expected from overt expressions. Finally, in a test of this experimentally, we showed that when neutral faces are psychophysically manipulated to alter their structure, color, and texture they yield similar patterns of impression biases, underscoring that each feature the algorithms used and learned to make accurate predictions was–at least in part–what was used by humans to arrive at similar judgements.

Study 1 introduced four machine learning models that were able to accurately predict emotion expressions from the structure, color, and texture of faces. These face metrics were selected to ensure that the models would be able to use low-level features to predict the expressive content of faces that were minimally- or non-expressive. That is, it was predicted that training models to use fundamental face metrics such as structure, color, and texture would create models more sensitive to the emotional content of faces expressing little or no emotion. All of the metric models performed with above chance levels of accuracy on a separate test set of expressions. Combining all three metrics into a single weighted model yielded the highest accuracy. The combined emotion recognition accuracy of these models was nearly 90%, statistically significantly higher than any of the three models individually, suggesting that each metric and their features uniquely contributed to performance of emotion recognition.

Study 1 also showed that there were structure, color, and texture differences across neutral faces that varied by gender. Overall, the results from Study 1 suggest that a more holistic view of person perception can be gained by examining individual face metrics/features as well as their combination. Male faces showed greater resemblance to power-oriented expressions (e.g., anger, happy) across each metric and female faces showed greater resemblance to fear expressions across each metric.

Study 2 revealed that structure, color, and texture resemblance to emotion expressions were related to human impressions in a similar gender-stereotypic manner: resemblance to anger and disgust expressions predicted power-oriented impressions, while resemblance to fear and surprise expressions predicted affiliative-oriented impressions. Similarly, the model output for anger expressions mediated the relationship between face gender and ratings of dominance, while human ratings of masculinity-femininity mediated the relationship between model outputs of anger/happy and dominance/trustworthiness, respectively.

Study 3 revealed that the emotion output from the trained machine learning models could be used to calculate higher-order impressions of neutral faces, and that these impressions were causally related to similar human judgements of the face. Specifically, algorithmically-derived dominance acted as the mediator between face gender and human ratings of dominance, and perceived gender acted as the mediator for both algorithmically-computed dominance/affiliation and human ratings of dominance/trustworthiness, respectively. In sum, Study 3 showed that dominance and affiliation could be reliably computed from the anger, disgust, fear, happy, sad, and surprise emotion output of the machine learning models, and that these algorithmically computed scores were related to similar human impressions through perceived face gender.

The results of Study 2 and 3 are important for several reasons. First, it showed that the emotion output from the models is meaningful and interpretable (i.e., not a “black box”). Second, it showed that humans are partially using emotion resemblance across all three face metric channels to make impressions of neutral faces. Having a tool that can predict subtle emotionality and impressions of neutral faces is an important tool for researchers and practitioners of affective science.

Lastly, Study 4 showed that when neutral faces were manipulated to resemble the structure, color, and texture of high and low dominance by high and low affiliation, anger, and happy expressions they were subsequently rated in a manner similar to faces naturally high/low in such attributes. These results provide the first experimental evidence showing that when faces are systematically manipulated to possess structure, color, and texture features of faces that incidentally or naturally have such features, they are judged in a similar, stereotypic manner. These results provide further evidence that individuals use fundamental face metrics–either separately or in combination–to make impression judgments of minimally- or non-expressive faces.

The results from Study 4 appear to be largest for quadrant I, II, anger, and happy transformed faces. This pattern of results is most likely due to both quadrants and both expressions being high dominant and high arousal, resulting in structure, color, and texture features that may be easier to identify. In conclusion, multiple comparisons between psychophysically manipulated and unaltered neutral faces support the primary hypothesis that face features the models learned are the same features that humans use to make impressions. These results experimentally replicated the correlational results reported in Studies 1 & 2.

Together, four studies highlight that while social visual perception at times can be accurate, emotion resembling features of the face can bias impressions and contribute to stereotypic evaluations (See Adams et al., 2017 for discussion). While there are myriad cues in the face beyond structure, color, and texture that can influence impressions, we believe that this is an important first step at disentangling the fundamental face metrics that appear to be influencing a perceivers' visual perception when making judgements. Despite not being able to specifically say what metrics are related to which impressions, the current results can definitely state that individuals appear to be using, at least partially, the structure, color, and texture cues related overt emotion expressions when judging others' faces.

The present results confirm that in addition to facial structure, color and texture related to emotion expressions are also important cues individuals use to make decisions. Research that has examined these cues in isolation demonstrated that they impact perceptions related to attractiveness and health (Pazda et al., 2016; Thorstenson et al., 2017; Perrett et al., 2020) as well as gender (Russell, 2003, 2009). It is certainly possible face color and texture influence impressions related to gender or health in a largely associative manner, much like how face structure has been shown to influence impressions via emotion overgeneralization (see, Heerey and Velani, 2010; Kocsor et al., 2019). That is, health influences mood (see, Yeung, 1996 for review), and therefore healthy individuals are likely associated with positivity. In so much as individuals are able to extract and associate specific color and texture cues with health, it may provide reciprocal feedback associations that can give rise to specific stereotypes. Indeed, neutral faces that were transformed to appear happier in Study 4 were rated as healthier, suggesting a correspondence between expressive cues across structure, color, and texture and health.

This work also suggests that computer vision techniques can be used to successfully extract and predict the emotional content of faces. Further, it was shown that machine learning models can be used to predict emotionality from minimally-expressive and non-expressive faces. Indeed, it appears as though fundamental face metrics, including structure, color, and texture, can be used to make meaningful predictions about such faces. Examining face metrics separately allows for parsing the relative (and combined) contribution each has to face perception.

### Reachable and Replicable Science

Machine learning is an important and rapidly expanding field of research within behavioral science. However, despite advances in the field it remains relatively inaccessible to non-programmers. Commercial applications have made strides in making machine learning programs straightforward for end-users by providing graphical user interfaces (GUIs) that allow for simple point-and-click operations (iMotions, 2019; Noldus Information Technology, 2019). However, most freely available and open source machine learning algorithms for person perception require some degree of coding or technological expertise. It is imperative that these tools and resources be available to all parts of the scientific community in order to advance research forward and answer both novel and old questions in new ways in a timely manner.

**Figure 6:**
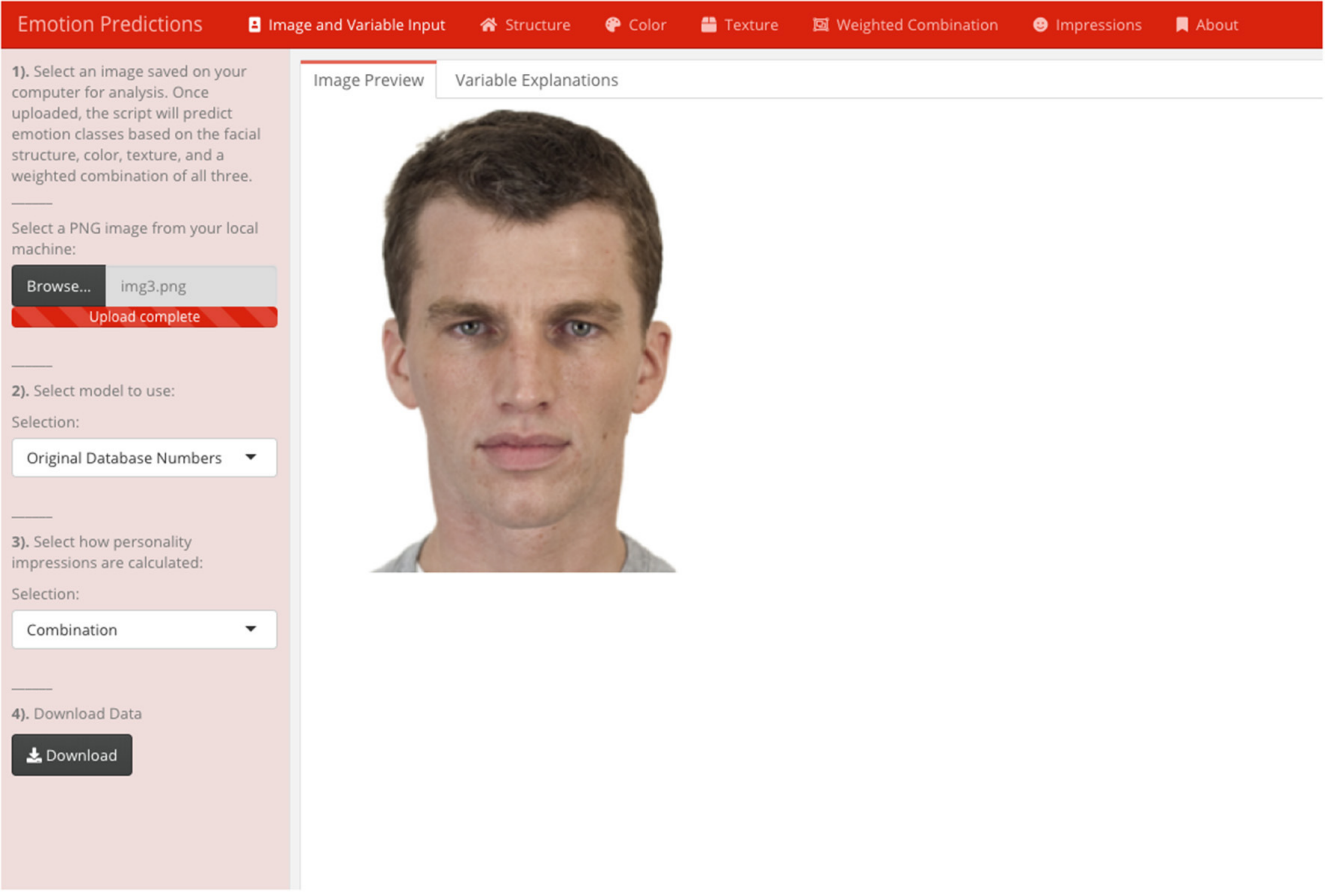
Image of GUI designed to predict the emotion expression and impressions of an uploaded face image. End-users have access to each feature model (structure, color, and texture), the weighted model of all three features, and algorithmically computed impressions for the uploaded face image (see panel at top of the GUI).

To this end, the current work includes an open source GUI (See Figure 6) written in R, JavaScript, and Python to utilize the structure, color, texture, and combined models in a point-and-click manner. The GUI is packaged as a shiny application residing in a Docker image, allowing for complete containerization (i.e., replicability across machines) so long as the end-user utilizes Docker on the host machine. The GUI allows for the user to easily upload pictures from their machine to be analyzed by each algorithm with moderate control over input parameters, such as the weight of each model in the aggregate predictive model. In addition to calculating expression estimates, the user is able to visualize each feature and obtain a computational estimate of where each face exists in the predicted two-dimensional social face space for every type of model. The user also has access to the computed data and can download it at any time straight from the GUI. All uploaded data to the app remains on the user's host machine, and no data is collected or stored by the app once it is shut down. Researchers can obtain access to this software by contacting the first author or by visiting https://www.daniel-albohn.com.

## Conclusions

It is a testament to the human visual system that individuals are able to derive meaningful information from the face given its complexity. Faces are important for social interactions, oftentimes signaling internal states and potential behavior through both numerous facial configurations as well as incidental resemblance to such features. Indeed, faces are so fundamental to forecasting intended and potential behavior that individuals effortlessly derive information from faces that only *incidentally* resemble emotion expression or personality traits. Non-expressive faces carry a surprising amount of information that aid individuals in forming impressions of others. Yet, despite the incredible amount of social information contained in neutral displays, relatively little work has utilized state of the art computer vision programs to reliably and accurately extract emotion expression from the face to make predictions about human behavior.

One central thesis put forth in the current research was that computer vision algorithms could be used to derive emotional content from minimally and non-expressive faces, and that the emotional content of these faces was related to human impressions. Results across four studies support these assumptions, revealing that not only can machine learning be used to accurately predict subtle emotion expressivity from neutral faces, but that these learned emotion outputs were related to human impressions in meaningful ways. Thus, the current work can begin to answer the question of what *exactly* are the mechanisms that influence an individual's impressions?

Beyond the utility of using machine learning algorithms to further our understanding of human perception, the current work also demonstrates–at a fundamental and objective level–that emotions are a powerful mechanism of impression formation. So much so, in fact, that in the face of no overt expressivity (i.e., a neutral face) humans appear to be grasping for any sort of emotional meaning in the face to make an informed decision, whether that be resemblance to emotion through such visual channels as structure, color, texture, or some combination of all three. The notion that emotions have such an impact on human impressions underscores the importance of understanding them within the broader context of person perception and non-verbal behavior.

## Data Availability Statement

The raw data supporting the conclusions of this article will be made available by the authors upon request, without undue reservation.

## Ethics Statement

The studies involving human participants were reviewed and approved by Office for Research Protections The Pennsylvania State University. Written informed consent for participation was not required for this study in accordance with the national legislation and the institutional requirements. Written informed consent was obtained from the individual(s) for the publication of any potentially identifiable images or data included in this article.

## Author's Note

DA is now at University of Chicago Booth School of Business.

## Author Contributions

DA conducted the research, performed the analyses, and wrote the initial draft of the manuscript under the supervision of RA. DA and RA both interpreted the results, revised and edited, commented on, and approved the final draft of the manuscript. All authors contributed to the article and approved the submitted version.

## Conflict of Interest

The authors declare that the research was conducted in the absence of any commercial or financial relationships that could be construed as a potential conflict of interest.
