# Supplementary material for: The Expressive Triad: Structure, Color, and Texture Similarity of Emotion Expressions Predict Impressions of Neutral Faces
Source: Front Psychol. 2021 Feb 25;12:612923. doi: 10.3389/fpsyg.2021.612923 (PMC7947284; doi:10.3389/fpsyg.2021.612923)
Supplement: Supplementary file 1 [file Data_Sheet_1.DOCX]

**Supplemental Materials 1**

**Study S1: Training the Structure, Color, and Texture Machine Learning Models**

**Model Training**

The purpose of Study S1 was to create a set of reliable subtle emotion expression detectors using low-level face features. Specifically, in Study S1 we trained three models using structure, color, and texture information from the internal portion of the face.

# Method

## Feature Extraction

Structure, color, and texture features were extracted from the internal portion of the face using an automatic pipeline developed specifically for the current research. The pipeline was first fed a three color channel face image that had been previously automatically centered and cropped to 200 x 200 pixels using the Viola and Jones algorithm (Viola and Jones, 2004). Next, the following steps were applied to each cropped face (see Figure 1, main text):

1. The cropped face is fitted with 68 facial landmarks using ensemble regression trees (Kazemi & Sullivan, 2014)
2. A polygonal face mask is applied to the face using the outermost of the 68 facial landmarks to isolate the internal portion of the face (i.e., removing background, hair, neck, etc.)
3. The extracted internal portion of the face is projected onto a size-standardized and transparent blank image to remove image background colors
4. **Structure features** are extracted by re-fitting the internal face with 68 facial landmarks (see Step 1)
5. **Color features** are extracted by obtaining the 3-channel color histogram with 32 bins per channel
6. The face is divided into a top image and a bottom image by approximately slicing it horizontally at the tip of the nose
7. Fourteen **texture features** (i.e., Haralick features) are extracted from both the top and bottom portion of the face by calculating the gray-level co-occurrence matrix averaged over each color channel (see, Haralick, Shanmugam, & Dinstein, 1973)

Upon successful completion, the pipeline outputs 68 pairs of shape features ([x, y] coordinates), 96 color features (32 * 3 color channels), and 24 texture features (14 top face features + 14 bottom face features).

Using the procedure defined above, features were extracted for several hundred faces per emotion expression from a variety of standardized research face databases, including FACES (Ebner, Riediger, & Lindenberger, 2010), NIMSTIM (Tottenham et al., 2009), Chicago Face Database (Ma, Correll, & Wittenbrink, 2015), RAFD (Langner, Dotsch, Bijlstra, Wigboldus, Hawk, & van Knippenberg, 2010), and Emotionet (Benitez-Quiroz, Srinivasan, Feng, Wang, & Martinez, 2017). These databases provide standardized, diverse (gender, race, and age), and high quality images to train the models. Table S1 reports the final number of emotion expressions for training and testing from which features were able to be extracted. It should be noted that several models were trained using a greater number of surprise expressions, but while these models nominally increased in training accuracy, their test accuracy dramatically dropped. This is likely due to the surprise face stimuli that were used to supplement the training set (i.e., from less standardized data sources). For this reason, only surprise expressions from experimentally validated data sources were used in the final model.

| **Train/Test** | **Anger** | **Disgust** | **Fear** | **Happy** | **Sad** | **Surprise** |
| --- | --- | --- | --- | --- | --- | --- |
| Train | 382 | 283 | 393 | 390 | 303 | 98 |
| Test | 168 | 120 | 171 | 167 | 129 | 31 |

***Table S1. Number of emotion expressions for training and testing.***

## Model Training

Each extracted feature was subjected to multiple different machine learning training algorithms to determine the best model using the H2o machine learning platform (LeDell et al., 2019) for R (R Core Team, 2019). Each feature was tested on a series of random forests, distributed randomized forests, generalized linear models (GLMs), various gradient boosting machines (GBMs), a default Deep Neural Net, a random grid of Deep Neural Nets, and two varieties of Stacked Ensembles (best of each family and all models combined) to determine the best model for learning. Grid search was used to select the optimal hyperparameters for each model. Stacked ensembles were trained with a GLM multinomial metalearner with elastic net regularization ($\alpha$ = 0.5, $\lambda$ = 5.586E-4). All models were trained with 10-fold cross-validation.

After training, optimal models were extracted and retained based on the highest training accuracy combined with lowest mean squared error (MSE) averaged across the 10-fold cross-validations. Top performing models and associated statistics for each feature are presented in Table S2.

| **Feature** | **Model Type** | **Training Accuracy** | **Training MPCE** | **Test Accuracy** | **Test MPCE** |
| --- | --- | --- | --- | --- | --- |
| Structure | GLM | 0.871 | 0.150 | 0.859 | 0.171 |
| Color | Stacked Ensemble | 0.544 | 0.459 | 0.553 | 0.466 |
| Texture | Stacked Ensemble | 0.652 | 0.358 | 0.698 | 0.314 |
| Combination | Weighted Combination | NA | NA | 0.892 | 0.121 |

***Table S2. Algorithms, accuracy, and mean per-class error (MPCE) for each face feature.***

The best performing structure model was a generalized linear model with $\alpha$ = 0.5 and $\lambda$ = 0.003229.

The best performing color model was a stacked ensemble model of all the trained models. This included 18 deep learning models with 1 to 4 hidden layers, two distributed random forest models with 44 and 48 trees, respectively, 33 GBMs with trees ranging from 34 to 82, and 1 GLM with ridge regularization ($\lambda$ = 0.008022).

The best performing texture model was a stacked ensemble model that contained the best model of each “family.” It consisted of a deep learning model with one hidden layer, two distributed random forest models both with 45 trees, two GBMs with 43 trees and 121 trees, and a GLM with ridge regularization ($\lambda$ = 0.00001805).

The weighted combination model was calculated by weighting each individual model’s output by its test performance accuracy. Test performance accuracy for each model was calculated via a separate set of faces that each model had never seen before (see Table S1 for details). Specifically, for each emotion, $i$, the weighted output, $\hat{y}_{k}$, was calculated as

$$\hat{y}_{k}=\sum_{i,j=1} \beta_{i}*M_{j}$$

whereby $\beta_{i}$ represents each model’s test accuracy (structure = 0.858779, color = 0.553435, and texture = 0.698473), and $M_{j}$ represents the model’s emotion expression outputs.

# Results

Model results are compared against chance level performance for a six item forced-choice test, or roughly 0.166. Test accuracy (as opposed to training accuracy) was used for each single sample t-test to obtain significance.

## Individual Feature Models

Ten-fold cross-validation accuracies and mean per-class errors are reported for each model in Table 2. Overall, accuracy for all of the models was significantly above chance, *t*(2) = 6.09, *p* = 0.026, 95% CIs [0.32, 1.08], *d* = 3.52. Individually, each model performed at above chance levels. Accuracy was highest for structure (86%), followed by texture (67%), and lastly color (58%) information.

## Combined Model

A weighted model was also created using the formula detailed in the Method. The weighted model takes into account the relative contribution (importance) of each feature on overall expression recognition. The combined, weighted model reached a test accuracy of 0.892, or nearly 3% higher than the highest performing individual metric model (structure).

To test whether the weighted model performed statistically better than the best performing individual metric model, two logistic regressions were modeled for comparison. The first model assessed the performance of the structure model by regressing individual stimulus accuracy on the intercept. Similarly, the second model assessed the performance of the weighted model by regressing individual stimulus accuracy on the intercept. Comparison of the two models using a Likelihood ratio test revealed that the weighted, combined model had significantly better performance than the top performing model, and by default any of the other individual models, $\chi^{2}$(0) = 101.49, *p* < .001.

## Discussion

In Study S1, three machine learning models were trained to predict emotion expressions using three separate low-level visual face metrics (structure, color, and texture) of expressive faces obtained from various databases (~300 per expression aside from surprise). First, a pipeline was developed to automatically extract each visual feature from only the internal portion of the face (i.e., no hair, clothing, etc.) to ensure that the models were learning to categorize using only internal face information and not other, extraneous factors. Next, features were simultaneously trained on many machine learning models. The highest achieving model for each was used to predict test accuracy on a separate set of faces (see, Van der Laan, Polley, & Hubbard, 2007). Additionally, a combined model was created using the weighted combination of all three metrics. All of the individual metric models performed significantly above chance levels of accuracy on the separate test set of expressions. Accuracy was highest for structure (86%), followed by texture (67%), and lastly color (58%) information. Critically, the weighted, combined emotion recognition model accuracy was nearly 90%, significantly higher than any of the three individual models suggesting that each feature uniquely contributed to overall performance.

**Supplemental Materials 2**

**Structure, Color, and Texture Individual Model Metric Comparison**

# Results

The output of each individual model was subjected to a linear mixed effects regression with fixed effects for face gender and predicted emotion expression (as computed by the relevant machine learning model), as well as the interaction between gender and emotion expression. Each model also included a random effect for each image. Reported results are from post hoc pairwise comparisons.


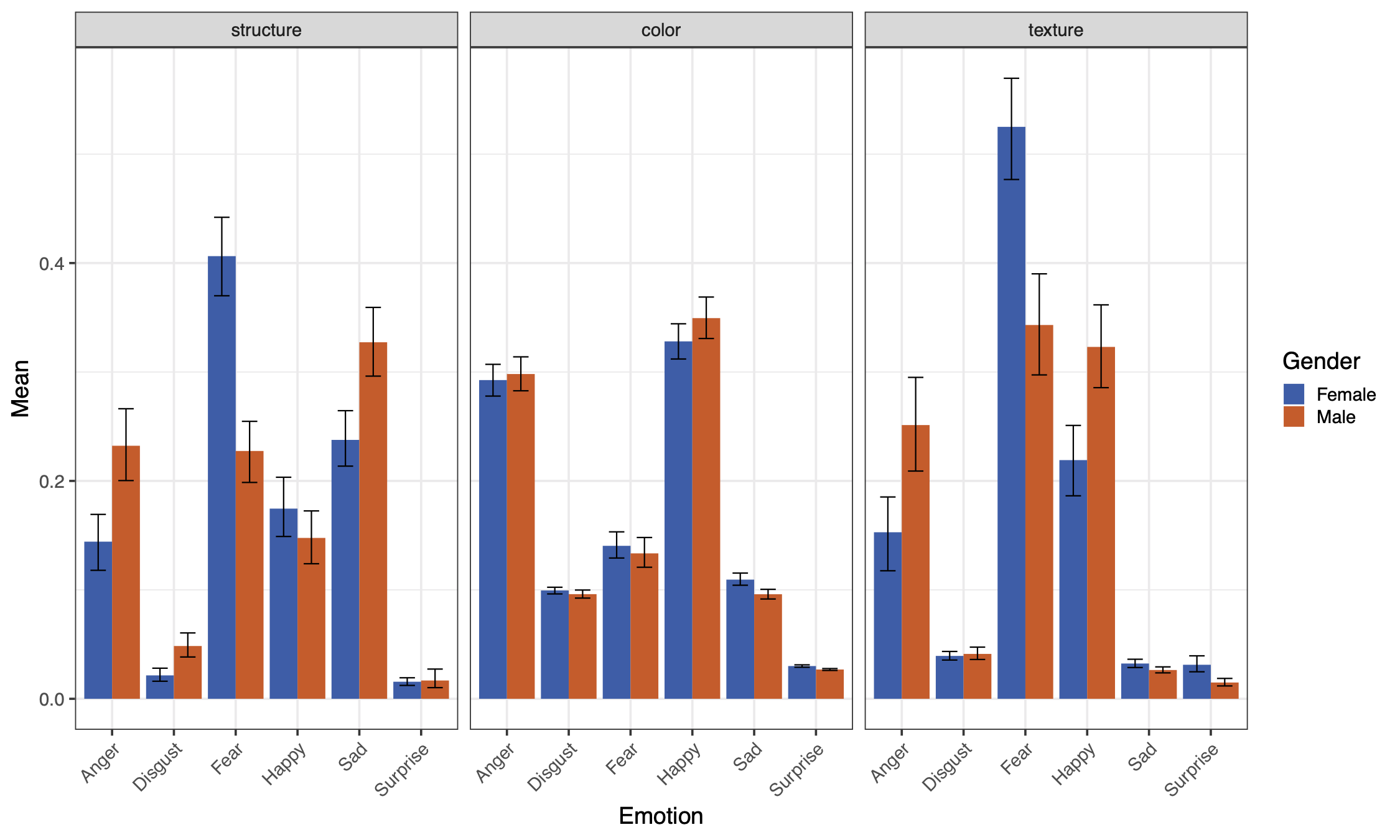


**Figure S1. Structure, color, and texture (panels) resemblance to each emotion by gender of neutral face. Error bars represent 95% confidence intervals.**

Overall, the structure, color, and texture differences for each emotion expression by face gender resulted in the predicted pattern of results.

***Structure***

As predicted, the structure of male neutral faces resembled anger expressions more than female faces (*t*(1086) = 4.31, *p* < .001, CIs [-0.13, -0.05]), whereas female faces showed greater resemblance to fear than male faces (*t*(1086) = 8.08, *p* < .001, CIs [0.13, 0.21]). Female faces also structurally resembled sadness more than male faces (*t*(1086) = -4.11, *p* < .001, CIs [-0.13, -0.05]).

***Color***

The color of female faces was less similar to happy expressions compared to male faces (*t*(1086) = -4.06, *p* < .001, CIs [-0.07, -0.02]). Female neutral faces were more similar in color to sadness compared to male neutral faces (*t*(1086) = 2.01, *p* = 0.045, CIs [0, 0.05]). Lastly, female neutral face color was more similar to anger expression compared to male neutral faces, (*t*(1086) = 2.01, *p* = 0.045, CIs [0, 0.05]).

***Texture***

The texture of female faces resembled fear expressions more than male faces (*t*(1086) = 5.13, *p* < .001, CIs [0.09, 0.21]). Similarly, the texture of male faces resembled anger (*t*(1086) = -2.15, *p* = 0.032, CIs [-0.12, -0.01]) and happy (*t*(1086) = -3.5, *p* < .001, CIs [-0.16, -0.05]) expressions more than female faces.

**Supplemental Materials 3**

**Study 2 Full Linear Mixed-Effects Regression by Gender for the Structure Model**

|  | **Value** | | | |
| --- | --- | --- | --- | --- |
| *Predictors* | *Estimates* | *CI* | *p* | *df* |
| (Intercept) | 0.25 | 0.22 – 0.28 | **<0.001** | 1086.00 |
| Gender [Male] | 0.09 | 0.05 – 0.13 | **<0.001** | 1086.00 |
| emotion [Disgust] | -0.23 | -0.27 – -0.18 | **<0.001** | 1086.00 |
| emotion [Fear] | 0.03 | -0.02 – 0.07 | 0.232 | 1086.00 |
| emotion [Happy] | -0.16 | -0.20 – -0.12 | **<0.001** | 1086.00 |
| emotion [Sad] | 0.10 | 0.06 – 0.15 | **<0.001** | 1086.00 |
| emotion [Surprise] | -0.24 | -0.28 – -0.20 | **<0.001** | 1086.00 |
| Gender [Male] * emotion [Disgust] | -0.07 | -0.13 – -0.01 | **0.017** | 1086.00 |
| Gender [Male] * emotion [Fear] | -0.26 | -0.32 – -0.21 | **<0.001** | 1086.00 |
| Gender [Male] * emotion [Happy] | -0.12 | -0.18 – -0.06 | **<0.001** | 1086.00 |
| Gender [Male] * emotion [Sad] | -0.00 | -0.06 – 0.05 | 0.887 | 1086.00 |
| Gender [Male] * emotion [Surprise] | -0.10 | -0.15 – -0.04 | **0.002** | 1086.00 |
| **Random Effects** | | | | |
| σ^2^ | 0.02 | | | |
| τ_00_ _image_ | 0.00 | | | |
| N _image_ | 183 | | | |
| Observations | 1098 | | | |
| Marginal R^2^ | 0.515 | | | |

**Study 2 Full Linear Mixed-Effects Regression by Gender for the Color Model**

|  | **Value** | | | |
| --- | --- | --- | --- | --- |
| *Predictors* | *Estimates* | *CI* | *p* | *df* |
| (Intercept) | 0.29 | 0.27 – 0.30 | **<0.001** | 1086.00 |
| Gender [Male] | -0.02 | -0.04 – 0.00 | 0.089 | 1086.00 |
| emotion [Disgust] | -0.17 | -0.20 – -0.15 | **<0.001** | 1086.00 |
| emotion [Fear] | -0.15 | -0.17 – -0.13 | **<0.001** | 1086.00 |
| emotion [Happy] | 0.01 | -0.01 – 0.03 | 0.437 | 1086.00 |
| emotion [Sad] | -0.15 | -0.18 – -0.13 | **<0.001** | 1086.00 |
| emotion [Surprise] | -0.25 | -0.28 – -0.23 | **<0.001** | 1086.00 |
| Gender [Male] * emotion [Disgust] | 0.02 | -0.01 – 0.05 | 0.302 | 1086.00 |
| Gender [Male] * emotion [Fear] | 0.02 | -0.01 – 0.05 | 0.149 | 1086.00 |
| Gender [Male] * emotion [Happy] | 0.07 | 0.03 – 0.10 | **<0.001** | 1086.00 |
| Gender [Male] * emotion [Sad] | -0.00 | -0.04 – 0.03 | 0.827 | 1086.00 |
| Gender [Male] * emotion [Surprise] | 0.01 | -0.02 – 0.05 | 0.376 | 1086.00 |
| **Random Effects** | | | | |
| σ^2^ | 0.01 | | | |
| τ_00_ _image_ | 0.00 | | | |
| N _image_ | 183 | | | |
| Observations | 1098 | | | |
| Marginal R^2^ | 0.631 | | | |

**Study 2 Full Linear Mixed-Effects Regression by Gender for Texture Model**

|  | **Value** | | | |
| --- | --- | --- | --- | --- |
| *Predictors* | *Estimates* | *CI* | *p* | *df* |
| (Intercept) | 0.13 | 0.09 – 0.17 | **<0.001** | 1086.00 |
| Gender [Male] | 0.06 | 0.01 – 0.12 | **0.032** | 1086.00 |
| emotion [Disgust] | -0.08 | -0.14 – -0.03 | **0.005** | 1086.00 |
| emotion [Fear] | 0.47 | 0.41 – 0.52 | **<0.001** | 1086.00 |
| emotion [Happy] | 0.03 | -0.03 – 0.09 | 0.266 | 1086.00 |
| emotion [Sad] | -0.09 | -0.15 – -0.03 | **0.002** | 1086.00 |
| emotion [Surprise] | -0.09 | -0.15 – -0.04 | **0.002** | 1086.00 |
| Gender [Male] * emotion [Disgust] | -0.06 | -0.14 – 0.02 | 0.170 | 1086.00 |
| Gender [Male] * emotion [Fear] | -0.22 | -0.30 – -0.13 | **<0.001** | 1086.00 |
| Gender [Male] * emotion [Happy] | 0.04 | -0.04 – 0.12 | 0.337 | 1086.00 |
| Gender [Male] * emotion [Sad] | -0.07 | -0.15 – 0.01 | 0.105 | 1086.00 |
| Gender [Male] * emotion [Surprise] | -0.08 | -0.16 – 0.00 | 0.054 | 1086.00 |
| **Random Effects** | | | | |
| σ^2^ | 0.04 | | | |
| τ_00_ _image_ | 0.00 | | | |
| N _image_ | 183 | | | |
| Observations | 1098 | | | |
| Marginal R^2^ | 0.432 | | | |

**Study 2 Full Linear Mixed-Effects Regression by Gender for the Weighted, Combined Model**

|  | **Value** | | | |
| --- | --- | --- | --- | --- |
| *Predictors* | *Estimates* | *CI* | *p* | *df* |
| (Intercept) | 0.19 | 0.17 – 0.20 | **<0.001** | 2268.00 |
| Gender [Male] | 0.07 | 0.05 – 0.09 | **<0.001** | 2268.00 |
| emotion [Disgust] | -0.14 | -0.16 – -0.12 | **<0.001** | 2268.00 |
| emotion [Fear] | 0.19 | 0.17 – 0.21 | **<0.001** | 2268.00 |
| emotion [Happy] | 0.04 | 0.02 – 0.06 | **<0.001** | 2268.00 |
| emotion [Sad] | -0.05 | -0.07 – -0.03 | **<0.001** | 2268.00 |
| emotion [Surprise] | -0.16 | -0.18 – -0.14 | **<0.001** | 2268.00 |
| Gender [Male] * emotion [Disgust] | -0.06 | -0.09 – -0.03 | **<0.001** | 2268.00 |
| Gender [Male] * emotion [Fear] | -0.20 | -0.23 – -0.17 | **<0.001** | 2268.00 |
| Gender [Male] * emotion [Happy] | -0.04 | -0.07 – -0.01 | **0.008** | 2268.00 |
| Gender [Male] * emotion [Sad] | -0.04 | -0.07 – -0.01 | **0.011** | 2268.00 |
| Gender [Male] * emotion [Surprise] | -0.08 | -0.11 – -0.05 | **<0.001** | 2268.00 |
| **Random Effects** | | | | |
| σ^2^ | 0.01 | | | |
| τ_00_ _image_predicted_ | 0.00 | | | |
| N _image_predicted_ | 380 | | | |
| Observations | 2280 | | | |
| Marginal R^2^ | 0.510 | | | |

**Supplemental Materials 4**

**Additional Mediations and Correlations for the Structure, Color, and Texture Models for Study 2**

| **Mediation** | **Path Estimates (standardized)** | | | | **Indirect Effect** | |
| --- | --- | --- | --- | --- | --- | --- |
|  | a (se) | b (se) | c (se) | c` (se) | ab | 95% CIs |
| **Structure** |  |  |  |  |  |  |
| *Face gender → Anger output → Dominance Ratings* | .20** (.07) | .34*** (.07) | .36*** (.07) | .34*** (.07) | .07* | [.02, .12] |
| *Anger output → Masculine-Feminine → Dominance Ratings* | .28*** (.06) | .41*** (.06) | .40*** (.07) | .29*** (.06) | .11* | [.06, .18] |
| Face gender → Happy output → Trustworthy Ratings | -.12 (.07) | .11 (.07) | -.36*** (.07) | -.35*** (.07) | -.01 | [-.06, .01] |
| *Happy output → Masculine-Feminine → Trustworthy Ratings* | -.13^t^ (.07) | -.44*** (.07) | .15 (.07) | .09*** (.07) | .06^t^ | [0, .13] ^b^ |
| **Color** |  |  |  |  |  |  |
| Face gender → Anger output → Dominance Ratings | -.09 (.07) | 0 (.07) | .36*** (.07) | .36*** (.07) | 0 | [-.02, .01] |
| Anger output → Masculine-Feminine → Dominance Ratings | -.09 (.07) | .49*** (.06) | -.03 (.07) | -.01*** (.07) | -.04 | [-.11, .03] |
| *Face gender → Happy output → Trustworthy Ratings* | .18* (.07) | .14** (.07) | -.36*** (.07) | -.38*** (.07) | .02^t^ | [0, .07] ^b^ |
| *Happy output → Masculine-Feminine → Trustworthy Ratings* | .17** (.07) | -.48*** (.07) | .07 (.07) | .15*** (.07) | -.08* | [-.15, .-.02] |
| **Texture** |  |  |  |  |  |  |
| *Face gender → Anger output → Dominance Ratings* | .12^t^ (.07) | .14** (.07) | .36*** (.07) | .34*** (.07) | .02^t^ | [0, .14] ^b^ |
| *Anger output → Masculine-Feminine → Dominance Ratings* | .14^t^ (.07) | .48*** (.06) | .18** (.07) | .11*** (.06) | .07^t^ | [-.01, .14] |
| *Face gender → Happy output → Trustworthy Ratings* | .21** (.07) | -.12^t^ (.07) | -.36*** (.07) | -.33*** (.07) | -.03^t^ | [-.07, 0]^b^ |
| *Happy output → Masculine-Feminine → Trustworthy Ratings* | .23** (.07) | -.43*** (.07) | -.2** (.07) | -.1*** (.07) | -.01* | [-.16, -.04] |

Note: ^t^ p < .1, * p < .05, ** p < .01, *** p < .001; ^b^ These CIs include 0 due to rounding.

***Table S3. Standardized regression estimates for structure, color, and texture mediation models. Italicized mediation models are in the predicted direction and support our hypotheses. Note that some of the mediations for happy color/texture and trustworthy are (marginally) significant, but in the opposite direction than predicted. There still support our hypotheses related to the confounded nature of gender and emotion and is explained more thoroughly in the main text.***

**
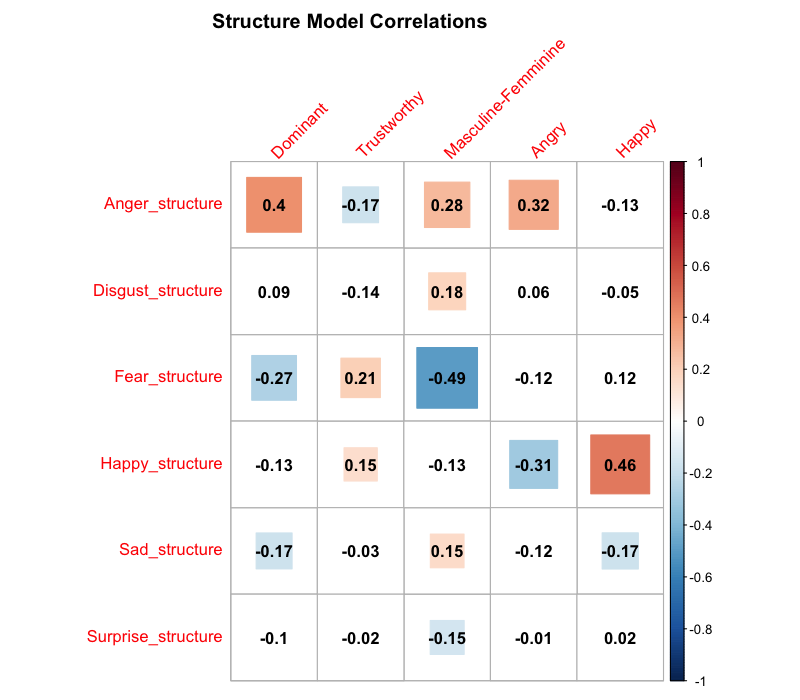
**

***Figure S2. Correlations between structure model predictions and human impressions. Machine-derived output is on the Y-axis, whereas human ratings are on the X-axis. Colored cells are significant. Hot colors are significant positive correlations and cool colors are significant negative correlations.***

**
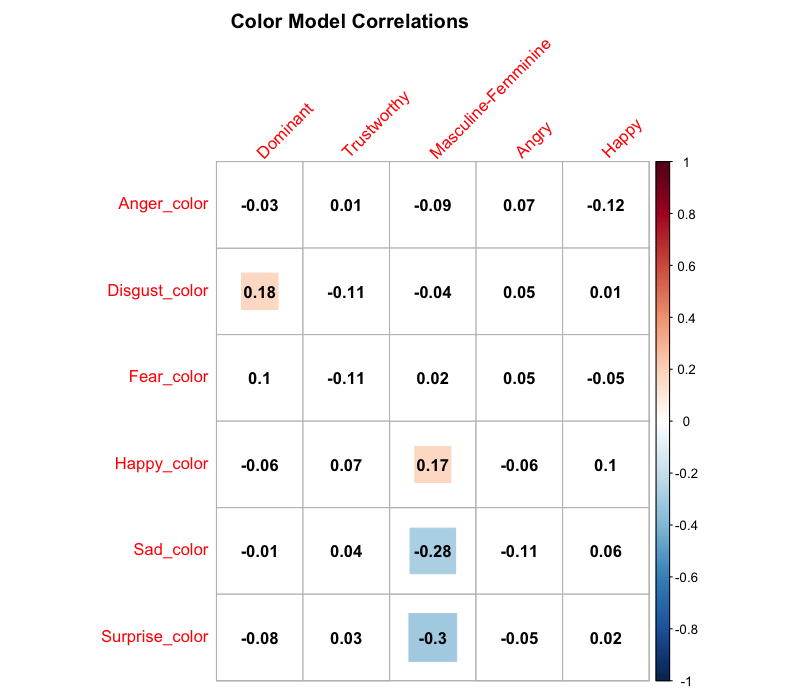
**

***Figure S3. Correlations between color model predictions and human impressions. Machine-derived output is on the Y-axis, whereas human ratings are on the X-axis. Colored cells are significant. Hot colors are significant positive correlations and cool colors are significant negative correlations.***

**
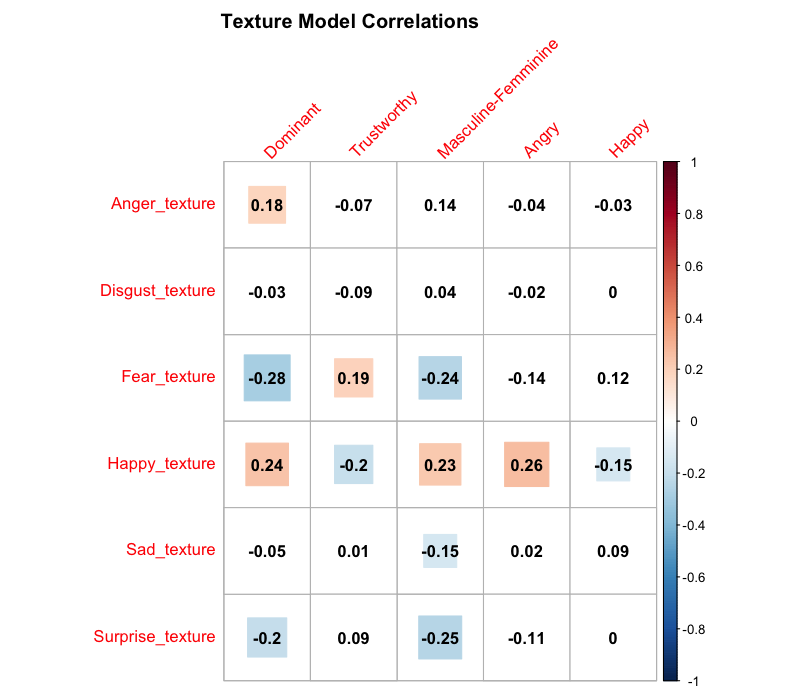
**

***Figure S4. Correlations between texture model predictions and human impressions. Machine-derived output is on the Y-axis, whereas human ratings are on the X-axis. Colored cells are significant. Hot colors are significant positive correlations and cool colors are significant negative correlations.***

**Supplemental Materials 5**

**Happy and Angry Psychophysical Face Transform Ratings**

**Results**

**Anger Transforms**

There was a main effect of rating, *F*(6, 18837.16) = 101.87, *p* < .001, 2= 0.46. No other main effects were significant. There was a significant interaction between face type and rating, (*F*(6, 18837.16) = 5.25, *p* < .001, 2= 0.02). Anger transform neutral faces were rated higher than close-to-origin neutral faces on dominance, estimate = 0.25, *SE* = 0.09, *t*(135.86) = 2.87, *p* = .005, CIs [0.08, 0.42], marginally higher in happiness, estimate = 0.14, *SE* = 0.09, *t*(135.86) = 1.67, *p* = .097, CIs [-0.03, 0.31], marginally higher in health, estimate = 0.18, *SE* = 0.09, *t*(135.86) = 2.06, *p* = .041, CIs [0.01, 0.35], and marginally higher in intelligence, estimate = 0.16, *SE* = 0.09, *t*(135.86) = 1.86, *p* = .065, CIs [-0.01, 0.33]. Anger transforms were also rated lower on babyishness, estimate = -0.21, *SE* = 0.09, *t*(135.86) = -2.41, *p* = .017, CIs [-0.38, -0.04].

There was also a significant interaction between rating and gender (*F*(6, 18837.16) = 21.98, *p* < .001, 2= 0.1). Female faces were rated higher than male faces on babyishness, estimate = 0.46, *SE* = 0.09, *t*(136.23) = 5.32, *p* < .001, CIs [0.29, 0.63], and trustworthiness, estimate = 0.09, *SE* = 0.09, *t*(136.23) = 1.04, *p* = .299, CIs [-0.08, 0.26]. Male faces were rated higher in dominance, estimate = -0.53, *SE* = 0.09, *t*(136.23) = -6.16, *p* < .001, CIs [-0.7, -0.36].

While there was no significant three-way interaction between face type, rating, and gender, *F*(6, 18837.16) = 1.68, *p* = .121, 2= 0.01, it should be noted that in the full model linear mixed-effects regression with planned contrasts several specific three-way interactions reached significance. Thus, pairwise comparisons were still examined for this three-way interaction. Female anger-transformed faces were marginally rated as *more* happy than close-to-origin female neutral faces, estimate = 0.23, *SE* = 0.12, *t*(133.56) = 1.89, *p* = .061, CIs [-0.01, 0.48]. On the other hand, male anger-transforms were rated as more dominant (estimate = 0.36, *SE* = 0.12, *t*(138.34) = 2.97, *p* = .004, CIs [0.12, 0.6]) and less babyish (estimate = -0.26, *SE* = 0.12, *t*(138.34) = -2.2, *p* = .03, CIs [-0.5, -0.03]) than close-to-origin neutral faces. With more power and specific *a priori*, planned contrasts these (and other) comparisons would have reached significance in the overall ANOVA model.

**Happy Transforms**

There was a main effect of rating, *F*(6, 16658.16) = 99.23, *p* < .001, 2= 0.49. No other main effects were significant. There was an interaction between face type and rating (*F*(6, 16658.16) = 6.07, *p* < .001, 2= 0.03). Happy transforms were rated higher than close-to-origin neutral faces on happiness, estimate = -0.27, *SE* = 0.09, *t*(115.6) = -3.1, *p* = .002, CIs [-0.45, -0.1], and intelligence, estimate = -0.16, *SE* = 0.09, *t*(115.6) = -1.86, *p* = .066, CIs [-0.34, 0.01]. Happy transforms were also rated lower on babyishness, estimate = 0.2, *SE* = 0.09, *t*(115.6) = 2.29, *p* = .024, CIs [0.03, 0.38].

There was also a significant interaction between rating and gender (*F*(6, 16658.16) = 13.16, *p* < .001, 2= 0.06). Female faces were rated higher than male faces on babyishness, estimate = 0.36, *SE* = 0.09, *t*(115.67) = 4.02, *p* < .001, CIs [0.18, 0.53], and male faces were rated higher in dominance, estimate = -0.45, *SE* = 0.09, *t*(115.67) = -5.07, *p* < .001, CIs [-0.62, -0.27].

Finally, there was a three-way interaction between face type, rating, and gender (*F*(6, 16658.16) = 2.61, *p* = .016, 2= 0.01). Happy transform female faces were rated as less babyish than close-to-origin neutral faces, estimate = 0.28, *SE* = 0.13, *t*(114.1) = 2.21, *p* = .029, CIs [0.03, 0.53]. Happy transform female faces were also rated as healthier (estimate = -0.3, *SE* = 0.13, *t*(114.1) = -2.42, *p* = .017, CIs [-0.55, -0.06]), more intelligent (estimate = -0.21, *SE* = 0.13, *t*(114.1) = -1.71, *p* = .09, CIs [-0.46, 0.03]), and more trustworthy (estimate = -0.22, *SE* = 0.13, *t*(114.1) = -1.73, *p* = .085, CIs [-0.47, 0.03]) than their close-to-origin counterparts. Happy transform male faces were only rated as slightly more dominant than close-to-origin neutrals, estimate = -0.23, *SE* = 0.12, *t*(117.29) = -1.85, *p* = .067, CIs [-0.48, 0.02]. Supplemental Materials 6 reports the full linear mixed-effects model.

**Supplemental Materials 6**

**Study 4 Full Linear Mixed-Effects Regression Estimates by Gender for Anger Transforms**

|  |  | | | |
| --- | --- | --- | --- | --- |
| *Predictors* | *Estimates* | *CI* | *p* | *df* |
| (Intercept) | 1.91 | 1.77 – 2.05 | **<0.001** | 248.17 |
| type [base] | -0.02 | -0.26 – 0.22 | 0.880 | 133.56 |
| block [babyish] | -0.12 | -0.25 – 0.00 | 0.056 | 18837.16 |
| block [dominance] | 0.03 | -0.10 – 0.15 | 0.684 | 18837.16 |
| block [happy] | -0.04 | -0.17 – 0.08 | 0.498 | 18837.16 |
| block [health] | 0.53 | 0.40 – 0.65 | **<0.001** | 18837.16 |
| block [smart] | 0.60 | 0.47 – 0.73 | **<0.001** | 18837.16 |
| block [trust] | 0.32 | 0.19 – 0.44 | **<0.001** | 18837.16 |
| gender [Male] | 0.08 | -0.08 – 0.23 | 0.336 | 132.70 |
| attractive | 0.18 | 0.17 – 0.20 | **<0.001** | 12293.69 |
| type [base] * block [babyish] | 0.17 | -0.09 – 0.43 | 0.203 | 18837.16 |
| type [base] * block [dominance] | -0.12 | -0.38 – 0.14 | 0.371 | 18837.16 |
| type [base] * block [happy] | -0.21 | -0.48 – 0.05 | 0.108 | 18837.16 |
| type [base] * block [health] | -0.16 | -0.42 – 0.11 | 0.245 | 18837.16 |
| type [base] * block [smart] | -0.16 | -0.42 – 0.10 | 0.230 | 18837.16 |
| type [base] * block [trust] | -0.11 | -0.37 – 0.15 | 0.416 | 18837.16 |
| type [base] * gender [Male] | -0.15 | -0.49 – 0.19 | 0.389 | 135.86 |
| block [babyish] * gender [Male] | -0.59 | -0.76 – -0.43 | **<0.001** | 18837.16 |
| block [dominance] * gender [Male] | 0.57 | 0.40 – 0.73 | **<0.001** | 18837.16 |
| block [happy] * gender [Male] | -0.15 | -0.32 – 0.02 | 0.075 | 18837.16 |
| block [health] * gender [Male] | 0.22 | 0.06 – 0.39 | **0.009** | 18837.16 |
| block [smart] * gender [Male] | 0.00 | -0.16 – 0.17 | 0.984 | 18837.16 |
| block [trust] * gender [Male] | -0.29 | -0.46 – -0.13 | **0.001** | 18837.16 |
| (type [base] * block [babyish]) * gender [Male] | 0.26 | -0.10 – 0.63 | 0.160 | 18837.16 |
| (type [base] * block [dominance]) * gender [Male] | -0.07 | -0.44 – 0.30 | 0.705 | 18837.16 |
| (type [base] * block [happy]) * gender [Male] | 0.33 | -0.04 – 0.69 | 0.080 | 18837.16 |
| (type [base] * block [health]) * gender [Male] | 0.14 | -0.22 – 0.51 | 0.450 | 18837.16 |
| (type [base] * block [smart]) * gender [Male] | 0.19 | -0.18 – 0.55 | 0.320 | 18837.16 |
| (type [base] * block [trust]) * gender [Male] | 0.41 | 0.04 – 0.77 | **0.030** | 18837.16 |
| **Random Effects** | | | | |
| σ^2^ | 1.90 | | | |
| τ_00_ _subject_nr_ | 0.27 | | | |
| τ_00_ _face_ | 0.02 | | | |
| ICC | 0.13 | | | |
| N _subject_nr_ | 216 | | | |
| N _face_ | 39 | | | |
| Observations | 19131 | | | |
| Marginal R^2^ | 0.100 | | | |

**Study 4 Full Linear Mixed-Effects Regression Estimates by Gender for Happy Transforms**

|  |  | | | |
| --- | --- | --- | --- | --- |
| *Predictors* | *Estimates* | *CI* | *p* | *df* |
| (Intercept) | 1.89 | 1.66 – 2.12 | **<0.001** | 143.39 |
| type [happy] | -0.18 | -0.43 – 0.06 | 0.146 | 114.10 |
| block [babyish] | 0.05 | -0.18 – 0.27 | 0.686 | 16658.16 |
| block [dominance] | -0.09 | -0.32 – 0.13 | 0.419 | 16658.16 |
| block [happy] | -0.26 | -0.48 – -0.03 | **0.025** | 16658.16 |
| block [health] | 0.37 | 0.15 – 0.60 | **0.001** | 16658.16 |
| block [smart] | 0.44 | 0.21 – 0.67 | **<0.001** | 16658.16 |
| block [trust] | 0.21 | -0.02 – 0.43 | 0.072 | 16658.16 |
| gender [Male] | -0.10 | -0.41 – 0.20 | 0.517 | 114.94 |
| attractive | 0.19 | 0.17 – 0.20 | **<0.001** | 10147.82 |
| type [happy] * block [babyish] | -0.09 | -0.35 – 0.17 | 0.479 | 16658.16 |
| type [happy] * block [dominance] | 0.30 | 0.04 – 0.56 | **0.022** | 16658.16 |
| type [happy] * block [happy] | 0.56 | 0.30 – 0.82 | **<0.001** | 16658.16 |
| type [happy] * block [health] | 0.49 | 0.23 – 0.75 | **<0.001** | 16658.16 |
| type [happy] * block [smart] | 0.40 | 0.14 – 0.66 | **0.003** | 16658.16 |
| type [happy] * block [trust] | 0.40 | 0.14 – 0.66 | **0.003** | 16658.16 |
| type [happy] * gender [Male] | 0.23 | -0.11 – 0.58 | 0.188 | 115.73 |
| block [babyish] * gender [Male] | -0.33 | -0.65 – -0.01 | **0.046** | 16658.16 |
| block [dominance] * gender [Male] | 0.49 | 0.17 – 0.82 | **0.003** | 16658.16 |
| block [happy] * gender [Male] | 0.18 | -0.15 – 0.50 | 0.285 | 16658.16 |
| block [health] * gender [Male] | 0.36 | 0.04 – 0.69 | **0.027** | 16658.16 |
| block [smart] * gender [Male] | 0.19 | -0.14 – 0.51 | 0.255 | 16658.16 |
| block [trust] * gender [Male] | 0.11 | -0.21 – 0.43 | 0.497 | 16658.16 |
| (type [happy] * block [babyish]) * gender [Male] | -0.08 | -0.45 – 0.28 | 0.655 | 16658.16 |
| (type [happy] * block [dominance]) * gender [Male] | -0.13 | -0.49 – 0.24 | 0.504 | 16658.16 |
| (type [happy] * block [happy]) * gender [Male] | -0.45 | -0.81 – -0.08 | **0.017** | 16658.16 |
| (type [happy] * block [health]) * gender [Male] | -0.56 | -0.92 – -0.19 | **0.003** | 16658.16 |
| (type [happy] * block [smart]) * gender [Male] | -0.33 | -0.70 – 0.03 | 0.075 | 16658.16 |
| (type [happy] * block [trust]) * gender [Male] | -0.45 | -0.81 – -0.08 | **0.017** | 16658.16 |
| **Random Effects** | | | | |
| σ^2^ | 1.86 | | | |
| τ_00_ _subject_nr_ | 0.29 | | | |
| τ_00_ _face_ | 0.02 | | | |
| ICC | 0.14 | | | |
| N _subject_nr_ | 216 | | | |
| N _face_ | 36 | | | |
| Observations | 16947 | | | |
| Marginal R^2^ | 0.093 | | | |

**Study 4 Full Linear Mixed-Effects Regression Estimates by Gender for Quadrant I Transforms**

|  |  | | | |
| --- | --- | --- | --- | --- |
| *Predictors* | *Estimates* | *CI* | *p* | *df* |
| (Intercept) | 1.87 | 1.65 – 2.10 | **<0.001** | 200.99 |
| type [quad1] | -0.19 | -0.42 – 0.04 | 0.110 | 159.40 |
| block [babyish] | 0.05 | -0.18 – 0.27 | 0.683 | 22183.79 |
| block [dominance] | -0.09 | -0.32 – 0.13 | 0.414 | 22183.79 |
| block [happy] | -0.26 | -0.48 – -0.03 | **0.024** | 22183.79 |
| block [health] | 0.37 | 0.15 – 0.60 | **0.001** | 22183.79 |
| block [smart] | 0.44 | 0.22 – 0.66 | **<0.001** | 22183.79 |
| block [trust] | 0.21 | -0.02 – 0.43 | 0.069 | 22183.79 |
| gender [Male] | -0.08 | -0.38 – 0.22 | 0.592 | 162.78 |
| attractive | 0.20 | 0.18 – 0.21 | **<0.001** | 14894.41 |
| type [quad1] * block [babyish] | -0.16 | -0.41 – 0.08 | 0.192 | 22183.79 |
| type [quad1] * block [dominance] | 0.29 | 0.04 – 0.53 | **0.022** | 22183.79 |
| type [quad1] * block [happy] | 0.51 | 0.26 – 0.75 | **<0.001** | 22183.79 |
| type [quad1] * block [health] | 0.41 | 0.17 – 0.66 | **0.001** | 22183.79 |
| type [quad1] * block [smart] | 0.28 | 0.03 – 0.52 | **0.027** | 22183.79 |
| type [quad1] * block [trust] | 0.41 | 0.17 – 0.66 | **0.001** | 22183.79 |
| type [quad1] * gender [Male] | 0.33 | 0.00 – 0.65 | 0.051 | 162.80 |
| block [babyish] * gender [Male] | -0.33 | -0.65 – -0.01 | **0.043** | 22183.79 |
| block [dominance] * gender [Male] | 0.49 | 0.18 – 0.81 | **0.002** | 22183.79 |
| block [happy] * gender [Male] | 0.18 | -0.14 – 0.50 | 0.279 | 22183.79 |
| block [health] * gender [Male] | 0.36 | 0.04 – 0.68 | **0.026** | 22183.79 |
| block [smart] * gender [Male] | 0.19 | -0.13 – 0.51 | 0.250 | 22183.79 |
| block [trust] * gender [Male] | 0.11 | -0.21 – 0.43 | 0.493 | 22183.79 |
| (type [quad1] * block [babyish]) * gender [Male] | -0.19 | -0.54 – 0.16 | 0.295 | 22183.79 |
| (type [quad1] * block [dominance]) * gender [Male] | -0.15 | -0.50 – 0.20 | 0.404 | 22183.79 |
| (type [quad1] * block [happy]) * gender [Male] | -0.59 | -0.94 – -0.24 | **0.001** | 22183.79 |
| (type [quad1] * block [health]) * gender [Male] | -0.65 | -1.00 – -0.30 | **<0.001** | 22183.79 |
| (type [quad1] * block [smart]) * gender [Male] | -0.43 | -0.78 – -0.07 | **0.017** | 22183.79 |
| (type [quad1] * block [trust]) * gender [Male] | -0.73 | -1.08 – -0.38 | **<0.001** | 22183.79 |
| **Random Effects** | | | | |
| σ^2^ | 1.82 | | | |
| τ_00_ _subject_nr_ | 0.28 | | | |
| τ_00_ _face_ | 0.02 | | | |
| ICC | 0.14 | | | |
| N _subject_nr_ | 216 | | | |
| N _face_ | 47 | | | |
| Observations | 22484 | | | |
| Marginal R^2^ | 0.096 | | | |

**Study 4 Full Linear Mixed-Effects Regression Estimates by Gender for Quadrant II Transforms**

|  |  | | | |
| --- | --- | --- | --- | --- |
| *Predictors* | *Estimates* | *CI* | *p* | *df* |
| (Intercept) | 1.90 | 1.66 – 2.13 | **<0.001** | 177.28 |
| type [quad2] | 0.01 | -0.24 – 0.25 | 0.963 | 141.38 |
| block [babyish] | 0.05 | -0.18 – 0.27 | 0.687 | 22652.59 |
| block [dominance] | -0.09 | -0.32 – 0.13 | 0.420 | 22652.59 |
| block [happy] | -0.26 | -0.48 – -0.03 | **0.026** | 22652.59 |
| block [health] | 0.37 | 0.15 – 0.60 | **0.001** | 22652.59 |
| block [smart] | 0.44 | 0.21 – 0.67 | **<0.001** | 22652.59 |
| block [trust] | 0.21 | -0.02 – 0.43 | 0.072 | 22652.59 |
| gender [Male] | -0.06 | -0.37 – 0.25 | 0.701 | 143.87 |
| attractive | 0.18 | 0.16 – 0.19 | **<0.001** | 15145.63 |
| type [quad2] * block [babyish] | -0.09 | -0.34 – 0.15 | 0.455 | 22652.59 |
| type [quad2] * block [dominance] | 0.09 | -0.16 – 0.34 | 0.462 | 22652.59 |
| type [quad2] * block [happy] | 0.13 | -0.12 – 0.38 | 0.314 | 22652.59 |
| type [quad2] * block [health] | 0.17 | -0.08 – 0.42 | 0.182 | 22652.59 |
| type [quad2] * block [smart] | 0.08 | -0.17 – 0.33 | 0.528 | 22652.59 |
| type [quad2] * block [trust] | 0.05 | -0.20 – 0.30 | 0.711 | 22652.59 |
| type [quad2] * gender [Male] | 0.05 | -0.30 – 0.39 | 0.797 | 143.86 |
| block [babyish] * gender [Male] | -0.33 | -0.65 – -0.01 | **0.046** | 22652.59 |
| block [dominance] * gender [Male] | 0.49 | 0.17 – 0.82 | **0.003** | 22652.59 |
| block [happy] * gender [Male] | 0.18 | -0.15 – 0.50 | 0.285 | 22652.59 |
| block [health] * gender [Male] | 0.36 | 0.04 – 0.69 | **0.028** | 22652.59 |
| block [smart] * gender [Male] | 0.19 | -0.14 – 0.51 | 0.256 | 22652.59 |
| block [trust] * gender [Male] | 0.11 | -0.21 – 0.44 | 0.498 | 22652.59 |
| (type [quad2] * block [babyish]) * gender [Male] | 0.02 | -0.33 – 0.38 | 0.899 | 22652.59 |
| (type [quad2] * block [dominance]) * gender [Male] | 0.16 | -0.20 – 0.51 | 0.387 | 22652.59 |
| (type [quad2] * block [happy]) * gender [Male] | -0.07 | -0.43 – 0.28 | 0.683 | 22652.59 |
| (type [quad2] * block [health]) * gender [Male] | -0.07 | -0.42 – 0.29 | 0.707 | 22652.59 |
| (type [quad2] * block [smart]) * gender [Male] | -0.08 | -0.44 – 0.27 | 0.652 | 22652.59 |
| (type [quad2] * block [trust]) * gender [Male] | -0.11 | -0.47 – 0.24 | 0.538 | 22652.59 |
| **Random Effects** | | | | |
| σ^2^ | 1.86 | | | |
| τ_00_ _subject_nr_ | 0.31 | | | |
| τ_00_ _face_ | 0.02 | | | |
| ICC | 0.15 | | | |
| N _subject_nr_ | 216 | | | |
| N _face_ | 48 | | | |
| Observations | 22953 | | | |
| Marginal R^2^ | 0.081 | | | |

**Study 4 Full Linear Mixed-Effects Regression Estimates by Gender for Quadrant III Transforms**

|  |  | | | |
| --- | --- | --- | --- | --- |
| *Predictors* | *Estimates* | *CI* | *p* | *df* |
| (Intercept) | 1.92 | 1.71 – 2.14 | **<0.001** | 260.57 |
| type [quad3] | 0.04 | -0.18 – 0.26 | 0.709 | 197.70 |
| block [babyish] | 0.05 | -0.18 – 0.27 | 0.683 | 21041.39 |
| block [dominance] | -0.09 | -0.32 – 0.13 | 0.414 | 21041.39 |
| block [happy] | -0.26 | -0.48 – -0.03 | **0.024** | 21041.39 |
| block [health] | 0.37 | 0.15 – 0.60 | **0.001** | 21041.39 |
| block [smart] | 0.44 | 0.22 – 0.66 | **<0.001** | 21041.39 |
| block [trust] | 0.21 | -0.02 – 0.43 | 0.068 | 21041.39 |
| gender [Male] | -0.09 | -0.37 – 0.19 | 0.540 | 201.42 |
| attractive | 0.17 | 0.16 – 0.18 | **<0.001** | 12680.56 |
| type [quad3] * block [babyish] | 0.09 | -0.16 – 0.34 | 0.469 | 21041.39 |
| type [quad3] * block [dominance] | 0.00 | -0.25 – 0.25 | 0.992 | 21041.39 |
| type [quad3] * block [happy] | 0.15 | -0.10 – 0.40 | 0.236 | 21041.39 |
| type [quad3] * block [health] | 0.10 | -0.15 – 0.35 | 0.434 | 21041.39 |
| type [quad3] * block [smart] | 0.01 | -0.24 – 0.26 | 0.961 | 21041.39 |
| type [quad3] * block [trust] | 0.07 | -0.18 – 0.32 | 0.599 | 21041.39 |
| type [quad3] * gender [Male] | 0.18 | -0.13 – 0.48 | 0.262 | 200.73 |
| block [babyish] * gender [Male] | -0.33 | -0.65 – -0.01 | **0.043** | 21041.39 |
| block [dominance] * gender [Male] | 0.49 | 0.18 – 0.81 | **0.002** | 21041.39 |
| block [happy] * gender [Male] | 0.18 | -0.14 – 0.50 | 0.279 | 21041.39 |
| block [health] * gender [Male] | 0.36 | 0.04 – 0.68 | **0.026** | 21041.39 |
| block [smart] * gender [Male] | 0.19 | -0.13 – 0.51 | 0.250 | 21041.39 |
| block [trust] * gender [Male] | 0.11 | -0.21 – 0.43 | 0.492 | 21041.39 |
| (type [quad3] * block [babyish]) * gender [Male] | -0.41 | -0.76 – -0.06 | **0.022** | 21041.39 |
| (type [quad3] * block [dominance]) * gender [Male] | 0.04 | -0.31 – 0.40 | 0.805 | 21041.39 |
| (type [quad3] * block [happy]) * gender [Male] | -0.47 | -0.82 – -0.12 | **0.009** | 21041.39 |
| (type [quad3] * block [health]) * gender [Male] | -0.29 | -0.65 – 0.06 | 0.101 | 21041.39 |
| (type [quad3] * block [smart]) * gender [Male] | -0.22 | -0.57 – 0.14 | 0.229 | 21041.39 |
| (type [quad3] * block [trust]) * gender [Male] | -0.39 | -0.74 – -0.03 | **0.032** | 21041.39 |
| **Random Effects** | | | | |
| σ^2^ | 1.82 | | | |
| τ_00_ _subject_nr_ | 0.31 | | | |
| τ_00_ _face_ | 0.01 | | | |
| ICC | 0.15 | | | |
| N _subject_nr_ | 216 | | | |
| N _face_ | 44 | | | |
| Observations | 21336 | | | |
| Marginal R^2^ | 0.078 | | | |

**Study 4 Full Linear Mixed-Effects Regression Estimates by Gender for Quadrant IV Transforms**

|  |  | | | |
| --- | --- | --- | --- | --- |
| *Predictors* | *Estimates* | *CI* | *p* | *df* |
| (Intercept) | 1.86 | 1.63 – 2.09 | **<0.001** | 186.19 |
| type [quad4] | -0.14 | -0.38 – 0.10 | 0.246 | 149.02 |
| block [babyish] | 0.05 | -0.18 – 0.27 | 0.685 | 21871.16 |
| block [dominance] | -0.09 | -0.32 – 0.13 | 0.418 | 21871.16 |
| block [happy] | -0.26 | -0.48 – -0.03 | **0.025** | 21871.16 |
| block [health] | 0.37 | 0.15 – 0.60 | **0.001** | 21871.16 |
| block [smart] | 0.44 | 0.22 – 0.67 | **<0.001** | 21871.16 |
| block [trust] | 0.21 | -0.02 – 0.43 | 0.071 | 21871.16 |
| gender [Male] | -0.07 | -0.38 – 0.24 | 0.654 | 151.68 |
| attractive | 0.20 | 0.18 – 0.21 | **<0.001** | 15044.25 |
| type [quad4] * block [babyish] | 0.12 | -0.13 – 0.36 | 0.353 | 21871.16 |
| type [quad4] * block [dominance] | 0.19 | -0.05 – 0.44 | 0.125 | 21871.16 |
| type [quad4] * block [happy] | 0.33 | 0.08 – 0.58 | **0.009** | 21871.16 |
| type [quad4] * block [health] | 0.38 | 0.13 – 0.63 | **0.003** | 21871.16 |
| type [quad4] * block [smart] | 0.34 | 0.09 – 0.59 | **0.007** | 21871.16 |
| type [quad4] * block [trust] | 0.33 | 0.08 – 0.57 | **0.010** | 21871.16 |
| type [quad4] * gender [Male] | 0.18 | -0.15 – 0.52 | 0.291 | 152.33 |
| block [babyish] * gender [Male] | -0.33 | -0.65 – -0.01 | **0.045** | 21871.16 |
| block [dominance] * gender [Male] | 0.49 | 0.17 – 0.82 | **0.003** | 21871.16 |
| block [happy] * gender [Male] | 0.18 | -0.15 – 0.50 | 0.283 | 21871.16 |
| block [health] * gender [Male] | 0.36 | 0.04 – 0.69 | **0.027** | 21871.16 |
| block [smart] * gender [Male] | 0.19 | -0.13 – 0.51 | 0.254 | 21871.16 |
| block [trust] * gender [Male] | 0.11 | -0.21 – 0.43 | 0.496 | 21871.16 |
| (type [quad4] * block [babyish]) * gender [Male] | -0.16 | -0.51 – 0.20 | 0.388 | 21871.16 |
| (type [quad4] * block [dominance]) * gender [Male] | -0.05 | -0.41 – 0.30 | 0.771 | 21871.16 |
| (type [quad4] * block [happy]) * gender [Male] | -0.39 | -0.75 – -0.04 | **0.030** | 21871.16 |
| (type [quad4] * block [health]) * gender [Male] | -0.43 | -0.79 – -0.08 | **0.017** | 21871.16 |
| (type [quad4] * block [smart]) * gender [Male] | -0.52 | -0.87 – -0.16 | **0.004** | 21871.16 |
| (type [quad4] * block [trust]) * gender [Male] | -0.55 | -0.90 – -0.19 | **0.002** | 21871.16 |
| **Random Effects** | | | | |
| σ^2^ | 1.85 | | | |
| τ_00_ _subject_nr_ | 0.28 | | | |
| τ_00_ _face_ | 0.02 | | | |
| ICC | 0.14 | | | |
| N _subject_nr_ | 216 | | | |
| N _face_ | 47 | | | |
| Observations | 22169 | | | |
| Marginal R^2^ | 0.087 | | | |

**References**

Benitez-Quiroz, C. F., Srinivasan, R., Feng, Q., Wang, Y., and Martinez, A. M. (2017). Emotionet challenge: Recognition of facial expressions of emotion in the wild. *arXiv preprint arXiv:1703.01210*.

Ebner, N. C., Riediger, M., and Lindenberger, U. (2010). FACES—a database of facial expressions in young, middle-aged, and older women and men: Development and validation. *Behavior Research Methods* 42, 351–362. doi:[*10.3758/BRM.42.1.351*](https://doi.org/10.3758/BRM.42.1.351).

Haralick, R. M., Shanmugam, K., and Dinstein, I. H. (1973). Textural features for image classification. *IEEE Transactions on systems, man, and cybernetics*, 610–621.

Kazemi, V., and Sullivan, J. (2014). One millisecond face alignment with an ensemble of regression trees. In the 2014 *IEEE conference on computer vision and pattern recognition* (Columbus, OH: IEEE), 1867–1874. doi:[*10.1109/CVPR.2014.241*](https://doi.org/10.1109/CVPR.2014.241).

LeDell, E., Gill, N., Aiello, S., Fu, A., Candel, A., Click, C., Kraljevic, T., Nykodym, T., Aboyoun, P., Kurka, M., et al. (2019). *H2o: R interface for ’h2o’*. Available at: [*https://CRAN.R-project.org/package=h2o*](https://cran.r-project.org/package=h2o).

Langner, O., Dotsch, R., Bijlstra, G., Wigboldus, D. H., Hawk, S. T., and van Knippenberg, A. (2010). Presentation and validation of the radboud faces database. *Cognition and emotion* 24, 1377–1388.

Ma, D. S., Correll, J., and Wittenbrink, B. (2015). The chicago face database: A free stimulus set of faces and norming data. *Behavior Research Methods* 47, 1122–1135. doi:[*10.3758/s13428-014-0532-5*](https://doi.org/10.3758/s13428-014-0532-5).

R Core Team (2019). *R: A language and environment for statistical computing*. Vienna, Austria: R Foundation for Statistical Computing Available at: [*https://www.R-project.org/*](https://www.r-project.org/).

Tottenham, N., Tanaka, J. W., Leon, A. C., McCarry, T., Nurse, M., Hare, T. A., Marcus, D. J., Westerlund, A., Casey, B., and Nelson, C. (2009). The NimStim set of facial expressions: Judgments from untrained research participants. *Psychiatry Research* 168, 242–249. doi:[*10.1016/j.psychres.2008.05.006*](https://doi.org/10.1016/j.psychres.2008.05.006).

Van der Laan, M. J., Polley, E. C., and Hubbard, A. E. (2007). Super learner. *Statistical applications in genetics and molecular biology* 6.

Viola, P., and Jones, M. J. (2004). Robust real-time face detection. *International journal of computer vision* 57, 137–154.
